# Characterization of Solid-State Complexities in Pharmaceutical Materials via Stimulated Raman Scattering Microscopy

**DOI:** 10.1021/acs.analchem.4c03163

**Published:** 2025-04-30

**Authors:** Elina A. Harju, Teemu Tomberg, Lea Wurr, Anneleen van Rijckeghem, Alba M. Arbiol Enguita, Vladimir Dordovic, Niklas G. Johansson, Heikki Räikkönen, Antti Isomäki, Jukka K. S. Saarinen, Keith C. Gordon, Bert van Veen, Clare J. Strachan

**Affiliations:** † Drug Research Program, Division of Pharmaceutical Chemistry and Technology, University of Helsinki, Helsinki 00014, Finland; ‡ Department of Chemistry, University of Helsinki, Helsinki 00014, Finland; § Zentiva, k.s., U.K.abelovny 130, Prague 10 10237, Czech Republic; ∥ Biomedicum Imaging Unit, University of Helsinki, Helsinki 00014, Finland; ⊥ Dodd-Walls Centre for Photonic and Quantum Technologies and Department of Chemistry, University of Otago, Dunedin 9054, New Zealand; # Orion Corporation, Espoo 02200, Finland

## Abstract

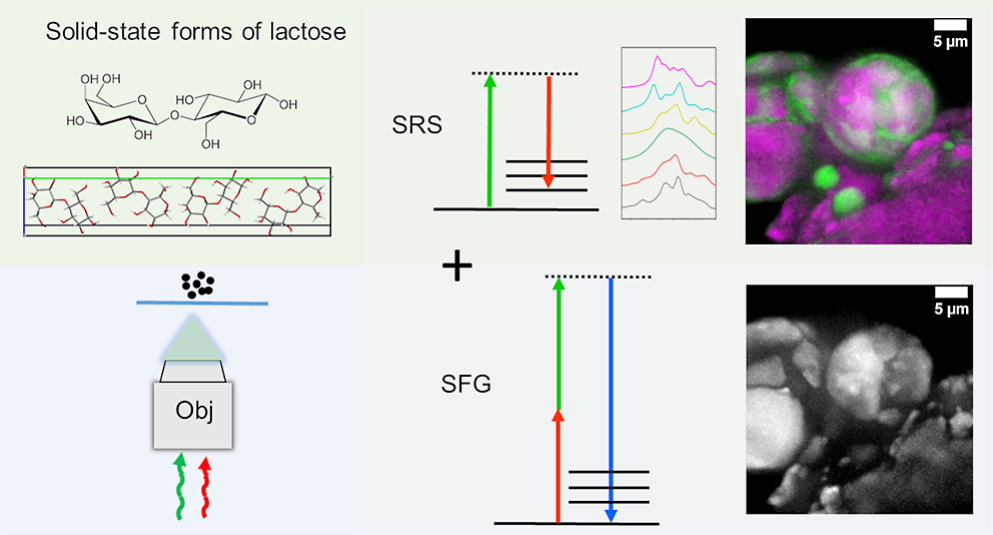

In this study, we employed stimulated Raman scattering (SRS) microscopy, augmented with sum frequency generation, to characterize complex solid-state mixtures, containing many solid-state forms of the same compound, for the first time. Five crystalline forms and one amorphous form of lactose were characterized and resolved, including two more recently defined anhydrous solid-state forms. Additionally, the complex solid-state character of several commercially available pharmaceutical tableting and inhalation grades of lactose was profiled. The advanced multimodal label-free microscopy method enabled visualization of the distribution of the solid-state forms with submicron spatial resolution, including the detection of trace levels. In addition, quantitative solid-state compositions of the lactose products were estimated. Overall SRS microscopy allows sensitive and specific spatially resolved solid-state characterization of complex mixtures, beyond what is possible with established (nonspatially resolved) characterization methods.

## Introduction

The solid-state landscape of molecular species can be complex, involving multiple crystalline polymorphs and more disordered amorphous and/or mesophase forms. The complexity expands further when other species are incorporated, to form, for example, solvates (including hydrates), cocrystals, salts, and coamorphous forms. With the solid-state form influencing important physiochemical properties, such as solubility and stability, its characterization is an integral component of, for example, the pharmaceutical and food industries. One of the main challenges in this field is solid-state analysis of complex heterogeneous samples, when multiple solid-state forms are present. Key issues in this respect—which remain largely unaddressed by conventional solid-state characterization methods—relate to the detection of trace levels of solid-state forms, analysis of degrees of disorder, quantification (especially when more than two solid-state forms are present), and distribution analysis (e.g., level of mixing, specific solid-state associations, surface enrichment).^1^

Raman spectroscopy is an analytical technique that uses scattered light to measure the vibrational energy modes of a sample. As light interacts with molecular vibrations, which alter the polarizability of molecule, the wavelength of the light is changed during the scattering, providing a molecular and structural fingerprint. Raman spectroscopy is a versatile tool for solid-state characterization, providing a fast, label-free, and nondestructive approach that requires minimal sample preparation and is sensitive to various solid-state forms. The most commonly used Raman spectroscopic technique is spontaneous Raman spectroscopy, where a single laser beam is used, and typically red-shifted Raman scattering (called Stokes-Raman scattering) is detected. However, the major challenge in spontaneous Raman spectroscopy is the low cross-section of Raman scattering, resulting in long acquisition times, particularly when it is used for mapping. To address this limitation, coherent Raman techniques, such as coherent anti-Stokes Raman scattering (CARS) and stimulated Raman scattering (SRS), have been developed.^2,3^ They utilize two spatially and temporally synchronized laser beams (pump and Stokes) which are tuned to match a Raman active molecular vibration of interest, providing coherent signal amplification. Unlike in spontaneous Raman spectroscopy, in CARS the detected Raman signal is blue-shifted (called anti-Stokes scattering). In SRS, the detection scheme is different, and changes in the laser beam intensities are monitored using a photodiode and a lock-in amplifier. Both coherent Raman techniques provide faster imaging with enhanced submicron spatial resolution and effectively circumvent the common issue of fluorescence background interference. Additionally, coherent Raman instruments typically enable integration of multiple imaging modalities, to provide complementary information. One particularly useful modality in solid-state characterization is sum frequency generation (SFG), which is characteristic of noncentrosymmetric crystalline materials.^4^ It is a second-order nonlinear optical process where the Stokes and pump photons convert into a new photon, the frequency of which is the sum of the input frequencies.

While the applicability of SRS microscopy in pharmaceutical solid-state characterization has been demonstrated,^5−7^ to our knowledge, the technique’s full potential for resolving and quantifying more than two solid-state forms simultaneously remains unexplored. In contrast to CARS, which has also been used to explore solid-states in pharmaceuticals applications,^8,9^ SRS offers several advantages. Its signal intensity is directly proportional to the concentration of the species of interest, facilitating quantitative analysis. Moreover, SRS avoids the nonresonant background interference typical in CARS, resulting in spectra that are more similar to those obtained through spontaneous Raman spectroscopy. Given all its advantages, SRS microscopy holds significant promise for advancing solid-state analysis across various fields, offering unique benefits over both traditional solid-state characterization methods and other Raman spectroscopy-based techniques.

Lactose (4-*O*-β-d-galactopyranosyl-d-glucopyranose, C_12_H_22_O_11_) is a disaccharide comprised of d-glucose and d-galactose linked by a β-1,4-glycosidic bond,^10^ and it serves as an ideal model for studying the solid-state complexity due to its relatively diverse solid-state landscape. Lactose can exist as either the α- or β-anomer, differentiated by the orientation of the hydroxyl group at the anomeric carbon, and has five widely accepted solid-state forms: amorphous lactose, α-lactose monohydrate (α-MNH), α-lactose anhydrous (α-ANH, stable triclinic form and more unstable monoclinic form) and β-lactose anhydrous (β-ANH).^11^ Moreover, two anhydrous crystal complex forms containing α- and β-lactose in a 1:1 ratio have been discovered and crystallographically characterized, namely a triclinic form^12^ (αβ_t_-ANH) and a more recently discovered monoclinic form^13^ (αβ_m_-ANH).

In the pharmaceutical and food industries, lactose is widely used in different kinds of products, with its solid-state form being an important aspect. In pharmaceuticals, lactose is commonly employed as a tableting agent or diluent, where it aids in the formation and stability of tablets.^14^ Different solid-state forms of lactose impact the disintegration behavior of a tablet which can alter subsequent dissolution and absorption of the drug.^15^ Additionally, lactose is used as a dry powder carrier in inhalers, facilitating the delivery of medication to the lungs. The interaction between lactose carrier and active pharmaceutical ingredient (API) is an important concept which could be affected by lactose solid-state form.

The European Pharmacopoeia, which sets quality standards throughout the pharmaceutical industry in Europe, outlines monographs for lactose monohydrate and anhydrous lactose, yet it does not enforce strict requirements for their solid-state nor anomeric composition. Typically, pharmaceutical-grade tableting lactose conforming to the monohydrate monograph includes minimal β-form impurities.^16^ These impurities may be present as distinct solid states or merged within α-MNH crystals, forming a solid solution.^17^ Also included in the lactose monohydrate monograph is spray-dried lactose containing both alpha monohydrate and amorphous lactose. Anhydrous lactose, preferred for moisture-sensitive APIs, typically has 70–80% anhydrous β-lactose and 20–30% anhydrous α-lactose.^14^ The existence of various solid-state forms, often not detailed in product descriptions, highlights a gap in the characterization of lactose.

In this study we investigated the potential of SRS microscopy to resolve the multiple solid-state forms of the model compound lactose and to explore the method’s applicability for qualitative and quantitative solid-state characterization of complex polycrystalline samples. Additionally, this paper provides insights into the solid-state characteristics of pharmaceutical lactose products used in tablet or inhalation formulations.

## Materials and Methods

### Materials

SuperTab 14SD, SuperTab 24AN, Lactohale 400 and Lactopress Granulated were obtained from DFE Pharma. Descriptions of the materials are provided in Table S1. The reference material for α-MNH was Pharmatose 200M (DFE Pharma). Methanol (anhydrous, max. 0.005% H_2_O, ≥99.8% purity, product code: 734030) and sodium hydroxide (technical grade, ≥97.0% purity, product code: 28240.292) were purchased from VWR International. The commercial lactose samples were stored in ambient conditions, in their original packaging, sealed with tape inside two plastic bags.

### Sample Preparation

The reference materials included amorphous spray-dried lactose (SD), α-MNH, the stable form of α-ANH, β-ANH, and a mixture of two crystalline forms in a 1:1 α/β ratio, referred to as αβ_t_-ANH/αβ_m_-ANH. Due to its extreme instability at relative humidities over 10%,^18−20^ the hygroscopic form of α-ANH could not be analyzed in the experimental conditions of this study and was excluded. Henceforth, “α-ANH” denotes the stable form.

The SD lactose, α-ANH, β-ANH and the αβ_t_-ANH/αβ_m_-ANH mixture were prepared from α-MNH. The protocol for preparing β-ANH was modified from a previously published protocol.^21^ A mixture of αβ_t_-ANH and αβ_m_-ANH was prepared following the protocol outlined by Nicholls et al. (2019), with minor modifications.^13^ The protocols for preparing all reference materials are described in detail in the Supporting Information. All reference materials were stored at room temperature in a desiccator containing silica gel.

For investigating the detectability of amorphousness in SuperTab 14SD and Lactohale 400, aliquots of those samples were stored at 57% relative humidity (RH) and 25 °C for 7 days to serve as negative controls, hereafter referred to as ‘conditioned samples’.

### SRS and SFG Imaging

SRS microscopy employing spectral focusing^22,23^ was performed using an in-house built instrument, described in detail elsewhere.^24^ Briefly, an Olympus FV3000 confocal laser scanning microscope (Olympus, Tokyo, Japan) was coupled with a dual-output ultrafast laser source (InSight X3+, Spectra-Physics, MKS Instruments, USA) providing a tunable pump beam and the fixed-wavelength Stokes beam at 1045 nm. The beams were passed through a Spectral Focusing, Timing and Recombination Unit (Newport SF-TRU, MKS Instruments, USA) before coupling to the microscope. A water immersion objective (Olympus UPLSAPO 60× W1600, NA 1.20) was used for focusing the light on the sample and the SRS signal was collected in the backscattered direction and measured with a large-area photodiode and lock-in amplifier (SRS Detection Set, APE Angewandte Physik & Elektronik, Berlin, Germany). The excitation beams were circularly polarized, and the backscattered signal passing through a linear polarizer was detected. Spectral scans were performed with the pump and Stokes laser wavelengths at 802 and 1045 nm, respectively. The spectral region between 2800–3000 cm^–1^ was measured with a 4 cm^–1^ step size by adjusting the temporal overlap of the Stokes and pump pulses. In the correlative SFG measurements, the Stokes and pump beams at the same wavelengths as for the SRS were used and the SFG signal was collected in the backscattered direction employing a nondescanned photomultiplier tube detector (PMT1001, Thorlabs, Germany).

In all imaging, the pixel size was set at 0.138 μm. For the reference materials, single *Z*-plane hyperspectral images were captured, whereas the commercial samples were measured as Z-stacks to capture depth information. For single-particle imaging, Z-slices were spaced 1 μm apart whereas for imaging of larger areas, a Z-slice interval of 3 μm was used.

### Analysis of SRS Images

For both qualitative and quantitative analyses, SRS images underwent several preprocessing steps, including min–max normalization, block-matching and 4D filtering (BM4D) for noise reduction,^25^ and baseline adjustment through minimum value subtraction. Subsequently, we utilized two supervised linear spectral decomposition methods (in a pixel-wise manner) implemented in an in-house developed MATLAB-based application: classical least-squares unmixing (CLS) and least absolute shrinkage and selection operator (LASSO), the latter one sourced from the publication by Lin et al. (2021).^26^ CLS represents a nonregularized approach, while LASSO introduces a regularization term that encourages sparsity in the solution by penalizing the magnitude of the coefficients.

### Characterization with Complementary Techniques

The α/β anomer contents of the lactose samples were analyzed using a previously reported solution-based ^1^H nuclear magnetic resonance (NMR) method.^27,28^ Moreover, the complementary characterization included X-ray powder diffraction (XRPD), scanning electron microscopy (SEM) and three different spontaneous Raman spectroscopy-based methods. Detailed descriptions of the methods can be found in the Supporting Information.

## Results and Discussion

### Characterization of Solid-State Forms of Lactose Reference Materials

XRPD patterns of the reference lactose materials revealed distinct peaks characteristic of each solid-state form (Figure S1). These patterns closely aligned with the predicted XRPD diffractograms (Figure S4) from the Cambridge Structural Database (CSD, version 2024.1). The corresponding CSD refcodes are BLACTO (β-ANH, monoclinic),^29^ EYOCUQ01 (α-ANH, triclinic),^30^ LACTOS11 (α-MNH, monoclinic)^31^ LAKKEO01 (αβ_t_-ANH, triclinic)^12^ and LAKKEO02 (αβ_m_-ANH, monoclinic).^13^ The β-ANH pattern also exhibited a reflection at 16.4° 2θ, suggesting contamination with α-MNH. The XRPD pattern of the SD sample presented a broad, almost featureless halo, indicative of amorphousness, but also revealed signs of trace crystallinity. This trace crystallinity was identified as αβ_t_-ANH based on peaks at 19.1° and 20.1° 2θ and the absence of the α-MNH peak at 16.4° 2θ (Figure S3). Previous studies have identified similar peaks in spray-dried lactose and attributed them to an α/β 5:3 solid-state form,^32,33^ which lacks definitive crystallographic evidence. The αβ_t_-ANH/αβ_m_-ANH reference mixture showed peaks characteristic to both forms, including the 19.1° and 20.1° 2θ reflections for αβ_t_-ANH and various other peaks characteristic to αβ_m_-ANH.

**Figure 1:**
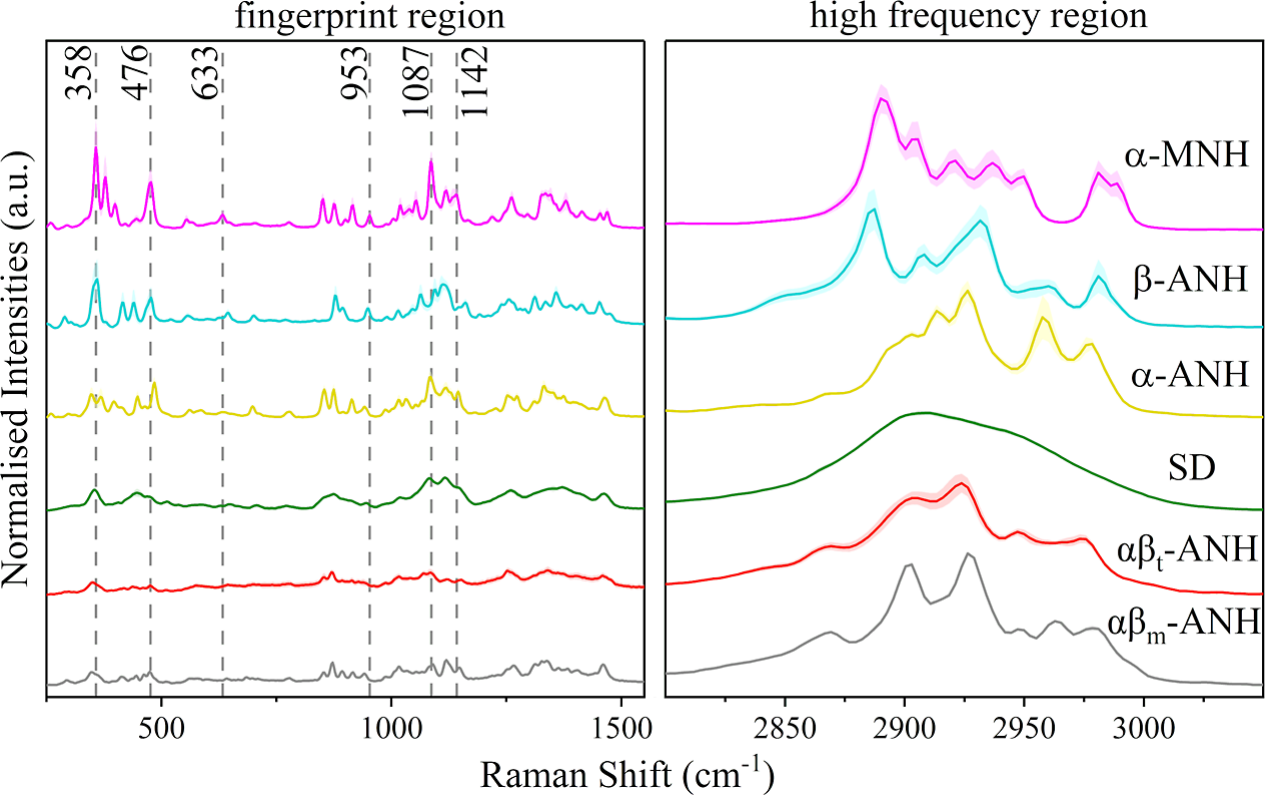
Spontaneous Raman spectra of the lactose reference materials measured with a confocal Raman microscope. Each spectrum represents an average from several randomly selected spots ±standard deviation. The dashed vertical lines indicate various peaks characteristic to α-MNH.

The spontaneous Raman microscopy spectra of the α-MNH, β-ANH, α-ANH and SD lactose exhibited distinct characteristics both in the fingerprint and high frequency regions (Figure 1). The highly ordered structure in the crystalline lactose samples leads to well-defined energy states and consequently produces sharp spectral lines, whereas amorphous materials typically give spectra with broader peaks. The vibrational modes of α-MNH within the fingerprint region have been described previously.^34^ One of the most prominent peaks in the Raman spectrum of α-MNH is the peak at 358 cm^–1^, which is attributed to ring torsions and deformation modes. The peaks at 476 cm^–1^ and 633 cm^–1^ correspond to deformations in the glycosidic linkage, while the 1087 cm^–1^ peak is indicative of stretching modes of C–C and C–O. Peaks at 953 and 1142 cm^–1^ highlight C–O bond stretching in the glycosidic linkage. The vibrational modes between 2800 to 3050 cm^–1^ are assigned to various stretching modes of CH and CH_2_ groups. The αβ_t_-ANH/αβ_m_-ANH mixture produced two distinct types of spectra, identified in SRS analyses as corresponding to each of the two forms. Despite the close structural resemblance between the αβ_t_-ANH and αβ_m_-ANH crystals,^13^ their Raman spectra can still be clearly differentiated. Notably, the Raman spectra of the two triclinic forms αβ_t_-ANH and α-ANH, exhibited strong similarities yet remain resolvable. Based on principal component analysis (PCA) (Figure S5), the CH stretching region emerges as a potential region in distinguishing among the samples and was chosen for the following SRS analyses.

The spontaneous Raman microscopy spectra of the crystalline form powders showed regional variation, which can be explained by the effect of crystal orientation. In Raman spectroscopy, the spectra are influenced by the polarization of the incident light relative to the crystal lattice, including the depolarization ratios of the molecular vibrations. This is due to the anisotropic nature of crystals, which leads to different polarizability depending on the crystal orientation. The orientation-dependency of fingerprint Raman spectra of α-MNH has been described before,^35^ but to our knowledge, this has not been investigated for all lactose solid-state forms and not for the high frequency spectral region. Notably, the SD sample showed the least regional variation due the isotropic nature of the amorphous form, which is reflected in the tight clustering observed in the PCA scores plots (Figure S5(b)).

**Figure 2:**
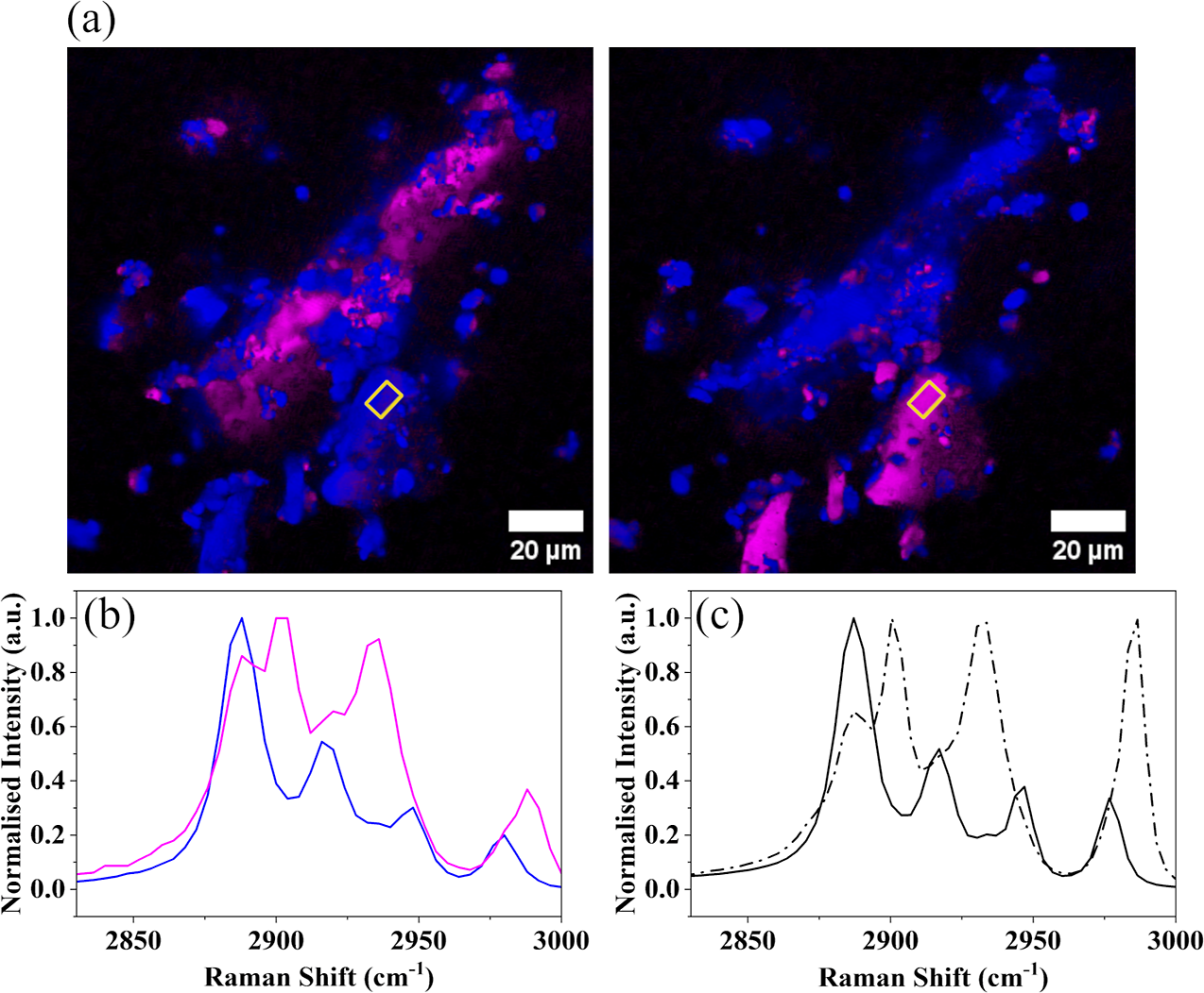
Orientation-dependency of Raman spectra of α-MNH. The same area of α-MNH was imaged with orthogonal polarizations, employing SRS imaging in transmitted direction (a); the yellow rectangle highlights the area from which average spectra, shown in (b), were extracted. The images in (a) were generated using CLS, employing the spectra in (b) as inputs. (c) Spontaneous Raman spectra of α-MNH obtained with parallel and perpendicular polarizations.

The polarization affects SRS spectra similarly to spontaneous Raman spectra, and we observed this especially with α-MNH. As the polarization effect introduced complexity into the multivariate data analysis of SRS images, we conducted polarized SRS^36^ experiments to investigate how crystal orientation influenced the SRS spectra. The reference materials were imaged using forward-detected SRS (f-SRS), with both the Stokes and pump beams polarized parallel in either the P or S direction (Figure 2a). The SRS orientation spectra were confirmed to be reasonable by comparison to polarized spontaneous Raman^37^ spectra (Figure 2c). Upon analyzing the SRS images, we found that, in practice, the spectra of each crystalline form could be well described by linear combinations of just two spectra (Table S3). Consequently, we chose to employ two reference spectra for each crystalline form in our analyses to adequately capture the orientation-related variations without complicating the model excessively. These reference spectra were selected from f-SRS images taken under P and S polarizations from particles which showed clear spectral differences between the two polarization states, as shown in Figure 2a. See section ‘Investigation of the polarization effect and its implications for data analysis’ in the Supporting Information for further details.

**Figure 3:**
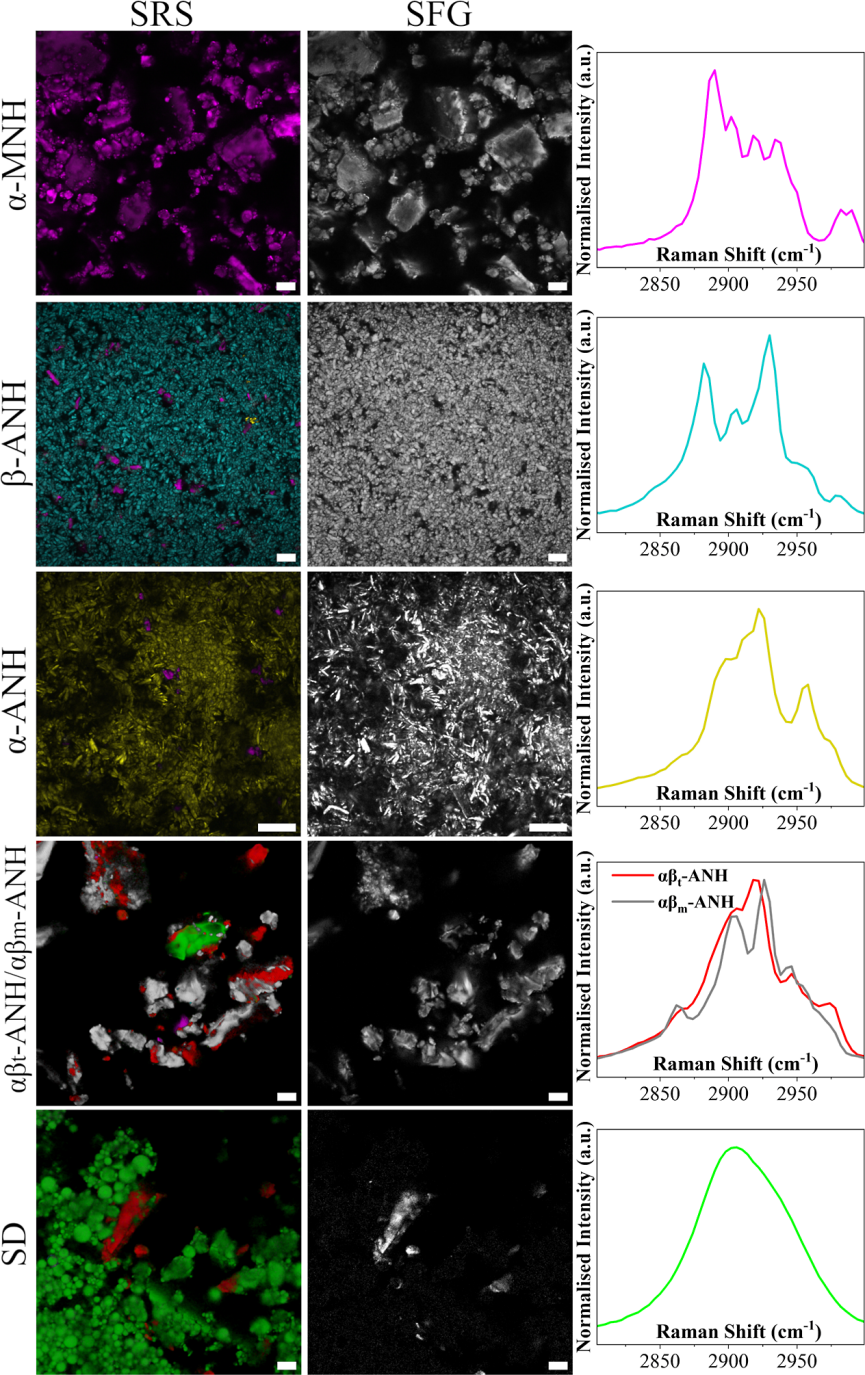
First column: false-colored SRS images (single plane) of the reference materials, generated with CLS and labeled according to the predominant form(s). Second column: correlative SFG images of the same areas. Scale bars, 10 μm. Third column: SRS spectra of each of the six forms.

The SRS images of the reference materials, produced using CLS analysis, alongside SRS spectra for each material, and correlative SFG images from the same areas are presented in Figure 3. The CLS analysis was performed using reference spectra from all six lactose forms to evaluate the purity of the reference materials. Apart from α-MNH, all reference materials showed evidence of contamination by other solid-state forms of lactose. Additionally, as expected from their noncentrosymmetric space groups, all crystalline forms exhibited SFG activity. With the SD reference sample, SFG detection proved to be particularly effective in detecting trace amounts of crystallinity, a capability similarly recognized by Schmitt et al. (2015).^38^ As explained earlier, this trace crystallinity was identified as αβ_t_-ANH based on the XRPD (Figure S3). For the αβ_t_-ANH/αβ_m_-ANH mixture, SFG provided additional specificity in identifying amorphous regions. Overall, the analysis of these reference materials demonstrates the advantage of SRS/SFG microscopy as a spatially resolved technique that enables selective spectral analysis of specific microscale regions within heterogeneous samples. The results for the NMR analysis of anomeric composition are presented in Table S2 and the NMR spectra in Figures S6–S10.

CLS unmixing of the SRS images of the reference materials successfully differentiated the solid-state forms, though some degree of overfitting was observed, especially in the case of the amorphous spectrum. The amorphous form displayed a broad spectral signature overlapping with the spectra of the crystalline forms, making it especially susceptible to being overfitted. In response to the challenges with spectral crosstalk and overfitting, LASSO regularized unmixing was adopted for all the commercial samples in this study, for both the qualitative and quantitative analyses. The lambda hyperparameter, which controls the level of sparsity in LASSO unmixing, was tuned to 0.1 for all SRS data, based on trace plots generated from ground truth images (i.e., images obtained from pure reference materials from areas visually assessed to be free of impurities) (Figure S21). This showed promise in simplifying the model by limiting the number of nonzero components per pixel.

The quantification of mixtures of different lactose solid-state forms was based on unmixing coefficients obtained from LASSO unmixing. Based on preliminary analysis of SRS image data (and XRPD), neither αβ_t_-ANH nor αβ_m_-ANH was detected in any of the commercial samples, and thus their spectra were not included as inputs for unmixing. Given the inherent differences in the polarizabilities of different lactose solid-state forms, scaling the input spectra differently for unmixing was deemed important. To approximate the Raman cross sections, all reference materials were measured with a time-gated Raman instrument that enabled a larger sampling volume (spot diameter 85 μm), thus averaging out some of the polarization-related variation (Figure S14). The anisotropic polarizability of each solid-state form was considered by scaling the two input spectra of each crystalline form based on their SRS spectra obtained with parallel and perpendicular polarizations, including scaling with polarization dependent detection efficiency of the SRS microscope.

### Characterization of Commercial Lactose Products

**Figure 4:**
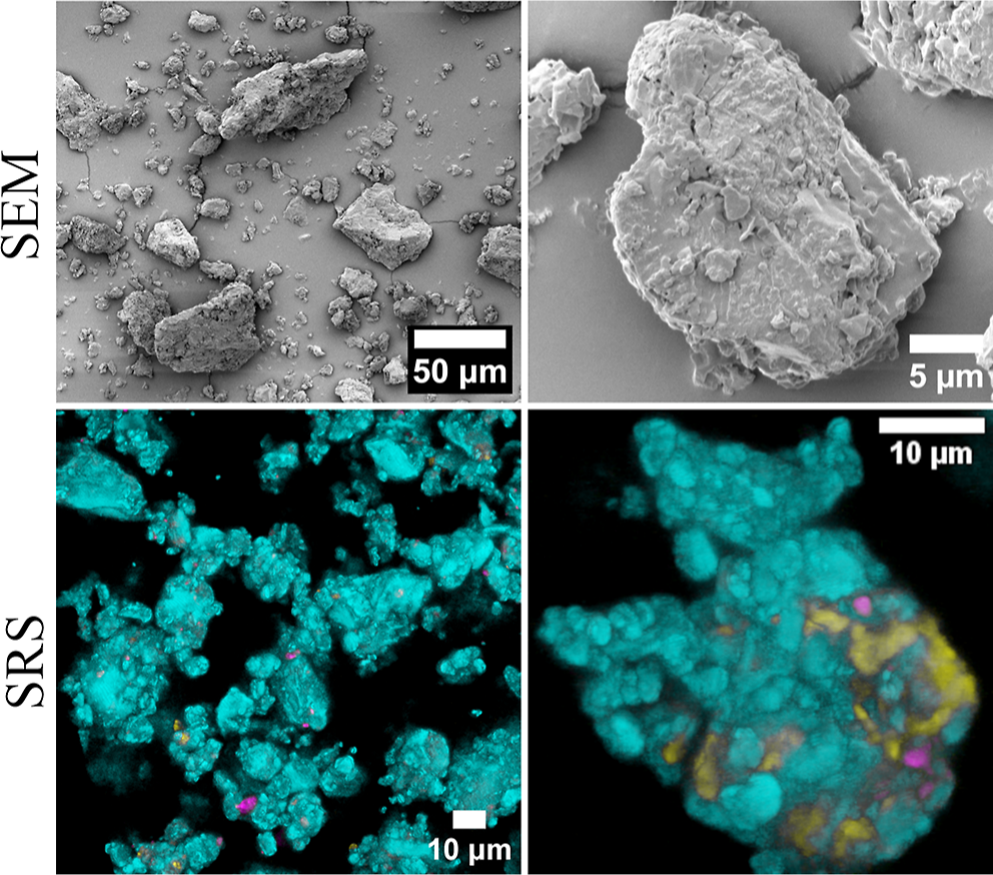
SEM and SRS characterization of Lactohale 400. The SRS images are maximum Z-projection overlays, where each color-coded channel indicates a different solid-state form of lactose: β-ANH (cyan), α-MNH (magenta) and α-ANH (yellow).

Lactohale 400, as specified by the manufacturer, is designed for dry powder inhalation (DPI) applications, and comprises anhydrous milled lactose with a median particle size of approximately 100 μm. According to the manufacturer specifications, the product is primarily composed of irregularly shaped particles, featuring a β-lactose anomer content of about 80%.^39^ The SEM and SRS analyses (Figure 4) concur with these characteristics, revealing a major presence of β-ANH and a smaller proportion of α-ANH. Moreover, trace amounts of α-MNH were detected. Spectra from the SRS images of the commercial samples are shown in Figures S23 and S24, and SRS characterization of SuperTab 24AN is shown in Figure S25.

The interaction between the carrier and the API, as well as particle size, are important aspects of DPI formulations. The adhesion between the API and lactose particles should be optimal – strong enough to maintain the mixture, yet weak enough to allow detachment when a patient inhales through the DPI device, creating an aerosol within the airstream. Particle size determines the number of sites available for API adherence. Moreover, the heterogeneity in particle size within lactose carrier is an interesting aspect of DPI formulations and the combination of smaller lactose fines with larger particles has been shown to improve DPI performance.^40,41^ A proposed mechanism suggests that fines initially occupy the higher energy binding sites on the carrier’s surface, which allows the API to bind to sites with lower adhesion strength, thus facilitating easier detachment during inhalation.^42^ This observation of fines on top of larger particles is evident in our SEM images (Figure 4). The SRS images (Figure 4) indicate that these smaller particles predominantly share the same solid-state form as their larger counterparts. Moreover, the milling process, aimed at reducing particle size to enhance API attachment, may cause surface amorphization, potentially impacting drug–carrier interactions and formulation stability.^43^ Such surface amorphousness might be very finely distributed and, in terms of our multimodal imaging approach, mixed with other lactose forms at a subpixel level. However, due to the issues with overfitting of the amorphous spectrum, we conclude that surface amorphousness in Lactohale 400, if present, could not be resolved from mixed pixels with high confidence based on visual inspection of the mixed pixel spectra. This outcome, coupled with the lack of positive controls in our experimental design, underscores the necessity for a more thorough investigation into the detectability of amorphousness in mixed pixels. While not pursued in this publication, the logical progression toward enhancing discrimination between amorphous and crystalline forms points toward integrating SRS and SFG data for multivariate unmixing in future work.

**Figure 5:**
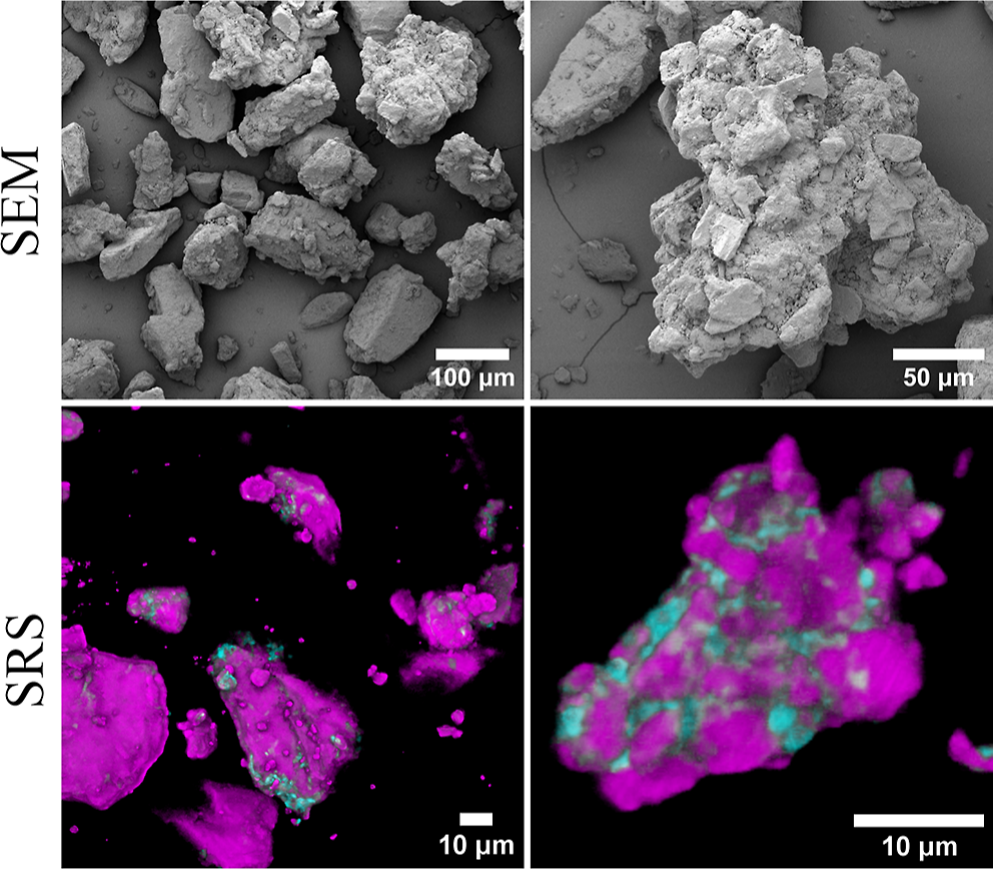
SEM and SRS characterization of Lactopress Granulated. The SRS images are maximum Z-projection overlays, where the cyan and magenta colors represent β-ANH and α-MNH, respectively.

Lactopress Granulated is produced by fluid-bed granulation of milled lactose monohydrate and is intended for direct compression applications. As per the product’s specifications, it is composed of α-MNH. Intriguingly, SRS analysis (Figure 5) of this sample not only confirmed the presence of α-MNH but also identified β-ANH. The β-ANH was observed in different forms, either evenly distributed around the α-MNH crystallites within the granulated particles or appearing as minor trace particles. The presence of β-ANH was confirmed by the XRPD data (Figure S22), where the reflection at 10.5° 2θ characteristic of the β-ANH was present in the diffractogram of the sample.

The presence and intragranular nature of the β-ANH may be critically important in making Lactopress Granulated directly compressible into tablets. This is because β-ANH is more compactible than α-MNH,^44−47^ meaning that the compacted mass maintains its cohesion without undergoing elastic recovery after the release of compaction pressure. Presumably, upon compression, it is the β-ANH between and around the α-MNH primary particles that undergo the required plastic deformation/brittle fracture for sufficient interparticulate bonding. The distribution of the β-ANH is likely to be a direct consequence of the wet granulation production method. During wet granulation, some of the lactose presumably dissolves and mutarotates to the β-anomer in solution. Through the formation of wet bridges, the solution agglomerates the suspended α-MNH primary particles. During drying, the dissolved lactose precipitates and forms solid bridges that bind the primary α-MNH particles together. This solidified lactose is partly composed of β-ANH.

SuperTab 14SD lactose is another direct compression grade lactose and is produced through a process where milled α-MNH is suspended in water, leading to a two-phase mixture of undissolved α-MNH particles and a dissolved lactose phase in anomer equilibrium.^45,48^ The spray-drying of this two-phase mixture leads to the formation of spherical agglomerates, where the undissolved α-MNH particles become embedded within a matrix of amorphous lactose, resulting from the rapid dehydration of the dissolved phase. The spherical morphology of the particles contributes to their good flow properties, an important attribute in the pharmaceutical industry.

**Figure 6:**
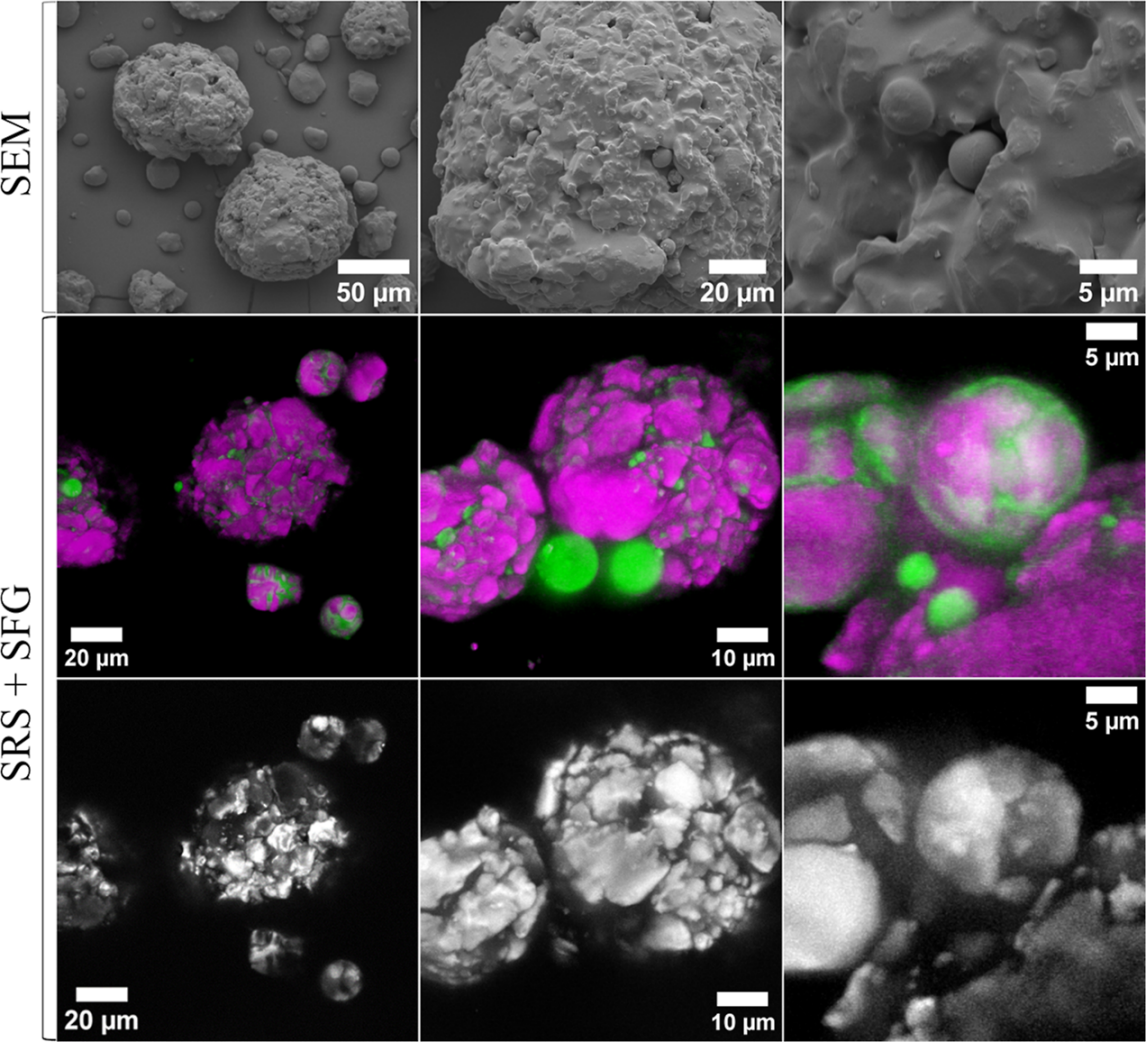
SEM, SRS and SFG images of SuperTab 14SD. The SRS and SFG images are correlative images from the same sample areas. The green and magenta colors represent amorphous lactose and α-MNH, respectively.

The series of SEM images (Figure 6) with increasing magnification reveal the morphological characteristics of SuperTab 14SD. We observed smaller, spherical particles with smooth surfaces, which were distributed among and embedded within larger spherical agglomerates. In contrast, the larger agglomerates displayed a more irregular surface topology. The SRS images (Figure 6), indicated that the smooth, small spheres in the SEM images were mostly amorphous lactose, while the larger agglomerates were mainly composed of α-MNH. The SFG signal specific to crystalline forms supports this interpretation.

Due to the poor compactability of α-MNH alone, the amorphous content in commercial spray-dried lactose is considered indispensable.^44−47^ It is responsible for the increased compactability as it adds a plastic deformation to the brittle fracture of crystalline lactose. Moreover, the amorphous content is believed to improve binding properties by crystallizing during compaction to form solid bridges. This is supported by the observation that preconditioning spray-dried lactose in high humidity, which turns the amorphous regions rubbery, leads to tablets with increased strength.^49^ However, preconditioning partially amorphous lactose in conditions that allows the amorphous content to crystallize results in tablets with decreased tensile strength and faster disintegration.^15^

### Quantitative Analysis

Quantification is one of the main challenges in solid-state analysis, especially when it comes to simultaneous quantification of multiple solid-state forms. From the pharmaceutical industry’s perspective, understanding the quantitative solid-state compositions of raw materials is important. Variability across different batches and manufacturers, along with solid-state conversions during storage and processing, can impact the performance of pharmaceutical products, which highlights the need for quantitative methods. To evaluate the potential of SRS for (semi)quantitative solid-state characterization, four pharmaceutical-grade lactose products (Lactohale 400, Lactopress Granulated, SuperTab 24AN and SuperTab 14SD) were examined, along with the conditioned SuperTab 14SD and Lactohale 400. Five Z-stacks were imaged at different sites of each sample, and the quantification based on area fractions and spectral analysis was explored. For both methods, all nonzero pixels were included in the analysis, with the exception that some isolated pixels (assumed to be noise) were removed prior to the quantification. This was done by applying a median filter with single pixel radius to binary masks generated from the images.

**Figure 7:**
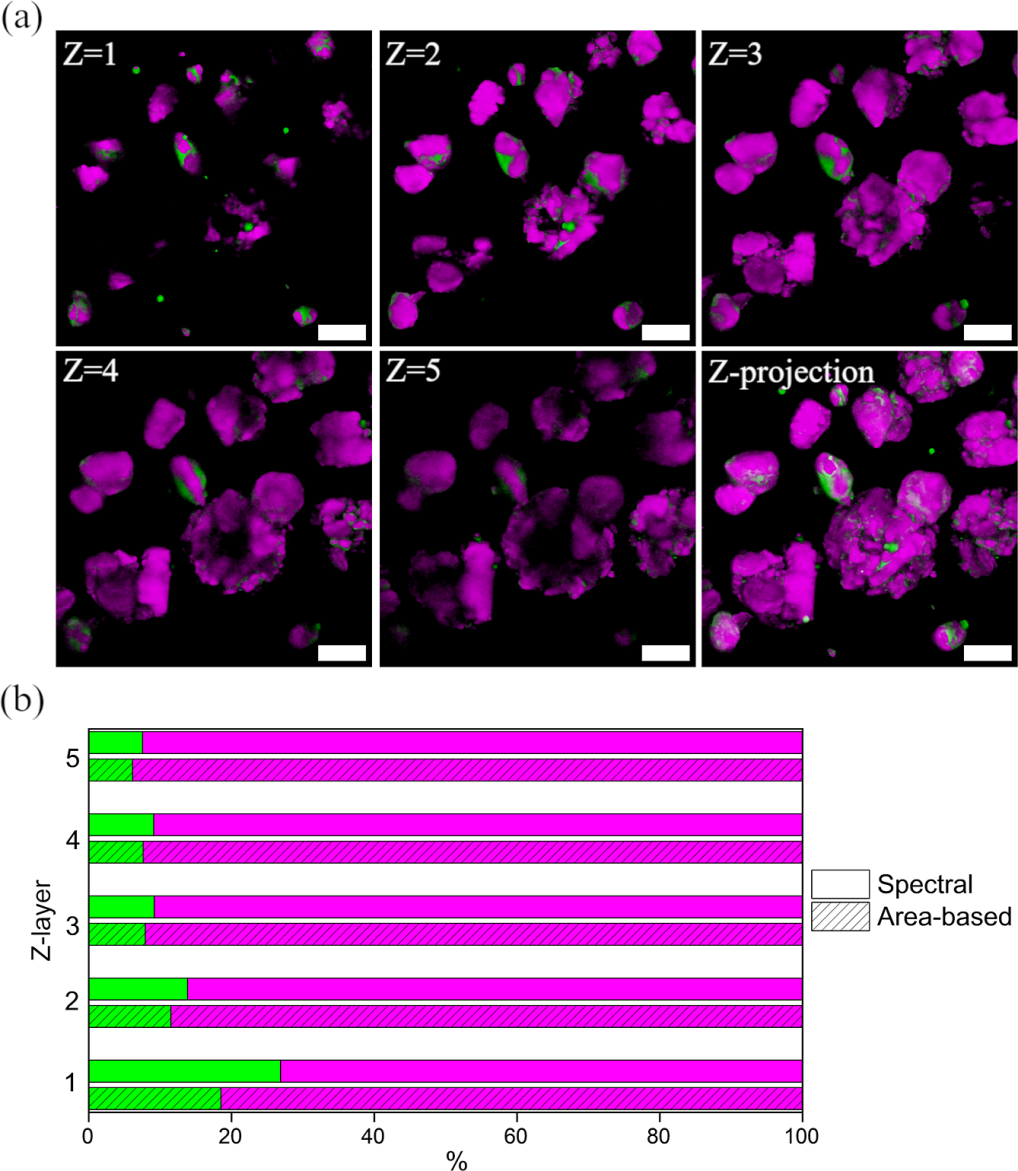
Example of the quantification of one SRS Z-stack of SuperTab 14SD sample. (a) SRS images from Z-levels 1–5 and the maximum Z-projection image of those. The spacing between adjacent Z-layers was 3 μm. Scale bars: 20 μm. (b) Area-based and spectral quantification, calculated for each Z-layer.

Figure 7 illustrates an example of the quantification of one SRS Z-stack of SuperTab 14SD sample, where the focusing depth was noticed to affect the quantification results. Specifically, different Z-layers often exhibited a trend, with the lowest layers (closest to the cover glass due to the use of inverted microscopy geometry) showing highest concentrations of minor compounds. This phenomenon can be attributed to several factors. In cases where minor lactose forms tend to adhere on the surfaces of larger particles, the first Z-layer provided the largest amount of exposed surface area where minor compounds were detectable. On the other hand, when minor forms were in the form of small individual particles, they would have settled at the bottom of the sample thus being more present on the first Z-layer. Conversely, Lactopress Granulated, for instance, where the minor forms appeared to be more evenly distributed around the particles, showed a different concentration pattern compared to SuperTab 14SD (Figure S26). While area-based quantification (Figure 7b) is commonly used for hyperspectral images, we considered this method less suitable for our data set due to the prevalence of mixed pixels. Consequently, spectral quantification was estimated to provide more accurate results for our imaging data.

The results from spectral quantification are presented in Table 1. In the evaluation of amorphous content, concentrations up to 1.8% per image were consistently detected in the conditioned samples, which should be purely crystalline. Given this, amorphous concentrations below 1.8% have to be interpreted as a potential false result and below the detection limit for this solid-state form. As creating subpixel level mixtures for experimentally determining limits of detection is not possible, we report limits of detection based on the signal-to-noise ratios of different components (Table S7, see section ‘Limit of detection’ in the Supporting Information for further details). Calibration measurements using physical mixtures of the four forms were conducted to support these findings, with results detailed in the section, ‘Quantitative analysis’, of the Supporting Information.

**Table 1:**
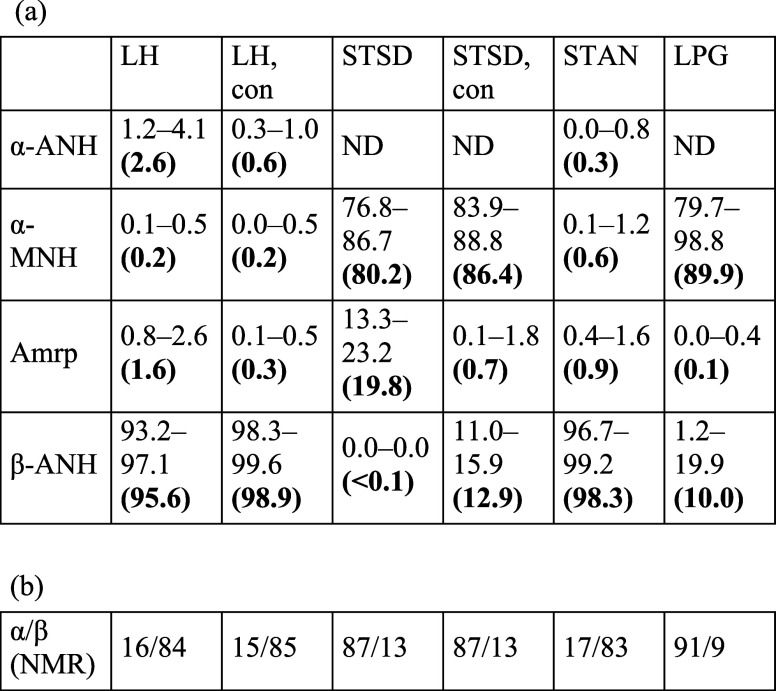
(a) Summary of SRS Quantification Results, Obtained with LASSO Unmixing; Values Represent the Range (Minimum–Maximum Concentration) across the 5 Z-Stacks; the Means Are Indicated in Bold in Parentheses; (b) Anomeric Compositions Obtained with NMR; See Table S6 for Results Obtained Using CLS Analysis^a^

a Abbreviations: LH = Lactohale 400; STSD = SuperTab 14SD; STAN = SuperTab 24AN; LPG = Lactopress Granulated; Amrp = amorphous; ND = not detected; con = conditioned.

The quantification results (Table 1) show that the two anhydrous lactose products, Lactohale 400 and SuperTab 24AN, contain more than 95% β-ANH. XRPD suggested the possible presence of α-MNH in Lactohale 400 through a very low intensity peak at 16.3° 2θ, with clearer traces in SuperTab 24AN (Figure S26). The SRS quantification for α-MNH exhibited a similar concentration trend. However, the SRS quantification (Table 1) and the XRPD peak intensities for α-MNH in the anhydrous lactose products were inconsistent with the NMR data, which revealed 16–17% α-anomer content. A similar case where the α-anomer was present without distinct crystal phases detectable in XRPD has been documented previously.^16^ Slight displacements observed in some of the XRPD reflections for the two anhydrous commercial samples (Figure S27) suggest the formation of a substitutional solid solution, where the α-anomer is incorporated into the β-ANH crystal structure as an impurity. Overall, these findings suggest that the SRS method presented in this paper exhibits greater sensitivity to variations in lactose solid-state forms than to differences in anomeric composition. In contrast to the anhydrous samples, the Lactopress Granulated results with XRPD, SRS and NMR methods were consistent with one another, with XRPD revealing traces of β-ANH (Figure S22) and SRS quantification confirming an average content of 10%, which aligned quite well with the NMR results. The NMR spectra of the commercial samples are presented in Figures S15–S20.

SuperTab 14SD showed an average content of 80.2% for α-MNH and 19.8% for amorphous lactose, the amorphous content being somewhat higher than previously reported at 13.4% with differential scanning calorimetry.^15^ Interestingly, both conditioned and untreated samples of SuperTab 14SD maintained the same anomeric ratio of 87/13, indicating that the conditioning did not induce epimerization. The larger increase in β-ANH content compared to the increase in α-MNH content suggested crystallization of the amorphous material mostly into β-ANH and, to a lesser extent, α-MNH. This, in turn, suggested that the amorphous content in SuperTab 14SD was predominantly composed of the β-anomer.

Quantification by (hyperspectral) microscopy exhibits some widely acknowledged fundamental challenges that are instructive to briefly discuss here (further explored in the section, ‘Quantitative analysis’, of the Supporting Information). Macro-scale solid-state composition quantification by microscopy suffers from limited sampling, where the captured images may not accurately represent the bulk composition. Segregation driven by differences in particle size and morphology can introduce systematic biases that cannot be resolved by increasing the measured area (Figure S34). Also, the dependency of quantification on spectral decomposition accuracy is an evident limitation, as overfitting was observed and balancing the regularization parameter is important (Figure S21 and S31). Despite the challenges, to our knowledge, this approach to quantifying solid-state forms has not been attempted before, offering new insights and a basis for further exploration in the field.

## Conclusions

In this study, we investigated the novel application of SRS microscopy to differentiate multiple solid-state forms of lactose, a model compound. To our knowledge, this was the first time as many as six solid-state forms of the same compound were simultaneously resolved based on their SRS spectra. Utilizing SRS imaging supported by complementary SFG, spontaneous Raman, XRPD, SEM, and NMR analyses, we provided new insights into the solid-state forms of lactose in both reference materials and commercially available products. SRS microscopy enabled fast, label-free solid-state characterization with high spatial resolution. This work highlights the potential of SRS microscopy as a powerful tool for evaluation of the solid-state compositions of complex polycrystalline samples. Despite some fundamental limitations in quantifying bulk materials, SRS microscopy excels in trace detection and in its ability to provide spatial information on form distribution. Moreover, the applicability of this method extends beyond the pharmaceutical industry, offering remarkable potential in fields such as materials science and food science, where understanding and controlling solid-state forms are equally critical.

## Supporting Information Available

Supporting Information Additional experimental details, materials, and methods, time-gated Raman, XRPD, NMR and SEM analysis results, PCA analysis of spontaneous Raman spectra, additional SRS results, investigation of the polarization effect and its implications for data analysis, investigation of the effect of regularization, additional experiments for quantitative SRS analysis (PDF)
