# Supplementary material for: Characterization of Solid-State Complexities in Pharmaceutical Materials via Stimulated Raman Scattering Microscopy
Source: Anal Chem. 2025 Apr 30;97(18):9627–37. doi: 10.1021/acs.analchem.4c03163 (PMC12079631; doi:10.1021/acs.analchem.4c03163)
Supplement: Supplementary file 1 — ac4c03163_si_001.pdf [file ac4c03163_si_001.pdf]

SUPPORTING INFORMATION

Characterization of solid-state complexities in pharmaceutical materials via stimulated Raman scattering microscopy

Elina A. Harju[†,*], Teemu Tomberg[†,‡], Lea Wurr[†], Anneleen van Rijckeghem[†], Alba M. Arbiol Enguita[†], Vladimir Dordovic[#], Niklas G. Johansson[†,‡], Heikki Räikkönen[†], Antti Isomäki[⊥], Jukka K. S. Saarinen[†], Keith C. Gordon[∥], Bert van Veen[§], Clare J. Strachan[†]

† Drug Research Program, Division of Pharmaceutical Chemistry and Technology, University of Helsinki, 00014 Helsinki, Finland

‡ Department of Chemistry, University of Helsinki, 00014 Helsinki, Finland

Zentiva, k.s., U Kabelovny 130, 10237 Prague 10, Czech Republic

⊥ Biomedicum Imaging Unit, University of Helsinki, 00014 Helsinki, Finland

∥ Dodd-Walls Centre for Photonic and Quantum Technologies and Department of Chemistry, University of Otago, Dunedin 9054, New Zealand

§ Orion Corporation, 02200 Espoo, Finland

*Corresponding author (elina.a.harju@helsinki.fi)

Table of contents

Table S1. Commercial lactose products S-4

Method descriptions S-4

Preparation of the lactose reference materials S-4

NMR analysis of anomeric composition S-4

X-Ray Powder Diffraction S-5

SEM S-5

Spontaneous Raman spectroscopy S-5

Complementary characterization of reference materials S-6

Figure S1. XRPD S-6

Figure S2. SEM S-7

Figure S3. XRPD pattern of the SD sample showing signs of trace crystallinity and the predicted XRPD diffractograms of $\alpha\beta_t$-ANH (LAKKEO01) and α-MNH (LACTOS11) S-8

Figure S4. Predicted XRPD diffractograms from Cambridge Structural Database S-9

Figure S5. Principal component analysis of spontaneous Raman microscopy spectra S-10
Table S2. Anomeric compositions of the reference materials, obtained with NMR S-11
Figure S6. ^{1}H NMR spectrum of α-MNH lactose S-11
Figure S7. ^{1}H NMR spectrum of β-ANH lactose S-12
Figure S8. ^{1}H NMR spectrum of α-ANH lactose S-12
Figure S9. ^{1}H NMR spectrum of $\alpha\beta_t/\alpha\beta_t$-ANH lactose S-13
Figure S10. ^{1}H NMR spectrum of SD lactose S-13
Investigation of the polarization effect and its implications for data analysis S-14
Table S3. Goodness of fit and residual analysis for different lactose forms using CLS S-14
Figure S11. Spectra obtained with the faster and extended approach S-15
Figure S12. SRS image of the lactose mixture containing β-ANH, α-ANH and α-MNH lactose and single-pixel spectra from the image S-16
Figure S13. SRS images of α-MNH, measured using either linearly or circularly polarized light S-17
Figure S14. Raman spectra of the reference materials, measured with the time-gated Raman instrument.... S-18
NMR spectra of the commercial samples S-19
Figure S15. ^{1}H NMR spectrum of Lactohale® 400 .... S-19
Figure S16. ^{1}H NMR spectrum of conditioned Lactohale® 400 .... S-20
Figure S17. ^{1}H NMR spectrum of Lactopress® Granulated S-20
Figure S18. ^{1}H NMR spectrum of SuperTab® 24AN S-21
Figure S19. ^{1}H NMR spectrum of SuperTab® 14SD S-21
Figure S20. ^{1}H NMR spectrum of conditioned SuperTab® 14SD S-22
Figure S21. Investigation of the effect of regularization S-23
Figure S22. XRPD diffractograms of the commercial lactose samples S-24
Figure S23. SRS characterization of Lactohale® 400 .... S-25
Figure S24. SRS characterization of Lactopress® Granulated S-26
Figure S25. SEM and SRS characterization of SuperTab® 24AN S-27
Figure S26. Example of the quantification of one SRS Z-stack of Lactopress® Granulated S-28
Figure S27. XRPD patterns of α-MNH, β-ANH, Lactohale® 400 and SuperTab® 24AN S-29
Quantitative analysis S-30
Calibration measurements S-30
Table S4. Mixture samples S-30
Figure S28. XRPD standard curves S-31
Table S5. Anomeric compositions of the mixture samples S-32
Figure S29. Calculated vs. measured anomeric compositions of the mixture samples. S-32
Figure S30. SRS images of the mixture samples S-34
Figure S31. Linear regression of SRS quantification vs. real compositions of the mixture samples S-35
CLS-based quantification of the commercial samples S-36

Table S6. Summary of SRS quantification results, obtained with CLS unmixing S-36
Limit of detection S-36
Figure S32. SRS image of the lactose mixture containing β-ANH, α-ANH, α-MNH, and SD lactose...... S-37
Table S7. Single pixel detection limit estimates for β-ANH, α-ANH, α-MNH, and amorphous lactose... S-38
Figure S33. SRS image of α-ANH showing trace particle of an unidentified contaminant S-38
Quantification error due to limited number of images S-39
Figure S34. Violin plots showing quantification results (area-based concentration) for 25 images recorded of the mixture samples #3 and #9 .... S-39
Figure S35. The change of standard deviation of the quantification results (sample #3) as function of number of images averaged S-40

Table S1. Commercial lactose products

Product	Lot, expiration date	Product description
SuperTab® 14SD	10B76ET, 11/2024	spray-dried α-MNH, especially for tablet and capsule formulations
SuperTab® 24AN	10BCCDP, 12/2024	anhydrous lactose for direct compression tablet applications
Lactohale® 400	10BH1CF, 2/2024	milled anhydrous lactose for dry powder inhalation applications
Lactopress® Granulated	106SFV5 , 11/2023	agglomerated α-MNH

Method descriptions

Preparation of the lactose reference materials

The protocol for preparing the β-ANH form was as follows: 10 mL of anhydrous methanol containing 20 mg of sodium hydroxide pellets and 1 g of α-MNH was stirred for 48 h at 20 °C. The slurry was filtered through a vacuum flask and washed several times with anhydrous methanol. The powder obtained was dried for 24 h in a vacuum oven at 60 °C. The α-ANH was prepared the same way as the β-ANH, but in this case no sodium hydroxide was used and the solution was stirred for 24 h. Amorphous lactose was prepared by spray drying lactose solution with a concentration of 18 g/L. A spray dryer, Buchi B290, was set up with an inlet temperature of 165 °C, outlet temperature of 90 °C, 30% flow and 100% aspirator. A mixture of $\alpha\beta_t$-ANH and $\alpha\beta_m$-ANH was prepared according to the following protocol: 40 g of α-MNH was dissolved in 100 mL of MilliQ water at 55 °C using a stirring hotplate to create a nearly saturated solution. The stirring lasted approximately two days, after which the suspension was sonicated to ensure complete dissolution. A 3 mL aliquot of the solution was transferred into a 5.5 cm diameter glass petri dish and dried in a fan oven at 120 °C for 90 minutes.

NMR analysis of anomeric composition

Circa 4 mg of each lactose sample was weighed into a Norell® Standard Series™ 5 mm NMR tube (Sigma-Aldrich, Saint Louis, MO, USA) and dissolved in 0.7 mL of dimethyl sulfoxide-d_6 (99.9 atom% D) containing trimethylsilane 0.03% (v/v) as an internal standard (Sigma-Aldrich, Saint Louis, MO, USA). The samples were vortexed for a few minutes until completely dissolved and directly submitted for NMR analysis. The NMR spectra were acquired at 298 K on a Bruker Ascend 400 MHz – Avance III HD NMR spectrometer (Bruker Corporation, Billerica, MA, USA), equipped with a 5 mm ^{1}H/BB double-resonance probe (Smartprobe™). Three subsequent ^{1}H NMR were run, recording 16 scans using a zg30 pulse sequence, and all the acquisitions finished within <15 minutes from addition of the solvent to each sample. All spectra were processed (phase and baseline correction) and analyzed using MestReNova software v14.1.0 (Mestrelab Research, Santiago de Compostela, Spain). Chemical shifts (δ) are reported in parts per million (ppm) relative to the residual internal standard at 0.00 ppm, showing the indicative peaks of β-lactose at ~6.6 ppm and α-lactose at ~6.3 ppm. To obtain the α/β anomeric ratio, the ratios of the integrated α and β peaks of each triplicate were compared.

X-Ray Powder Diffraction

X-ray powder diffraction (XRPD) measurements were conducted using a Malvern PANanalytical Empyrean diffractometer (Malvern PANanalytical, UK) equipped with a copper Kα radiation source, which was run under a voltage of 45kV and a current of 40mA. Samples were prepared by sandwiching them between two pieces of Kapton tape and analyzed in transmission mode. The diffraction scans were performed over a range of 10 to 35° 2θ , using a step size of 0.013° 2θ.

SEM

Scanning electron microscopy (SEM) was conducted using an FEI Quanta 250 Field Emission Gun SEM (FEI, USA). The samples were mounted on aluminum stubs and coated with a thin layer of platinum to enhance electrical conductivity. Images were taken under high vacuum conditions, using beam voltages in the range of 2–4 kV, and the spot size was set to 3 (arbitrary units).

Spontaneous Raman spectroscopy

Three different spontaneous Raman instruments were employed to address various aspects of the study. Firstly, a confocal Raman microscope (NT-MDT Ntegra Spectra, Russia) equipped with a 532 nm laser, power at the sample approximately 10mW, and a 100 × objective (Mitutoyo, Japan) was used to measure the Raman spectra of reference materials with back scattering geometry. The acquisition time was 2 seconds and spectra were accumulated over 10 scans.

Secondly, in order to approximate the Raman-cross sections of lactose forms, a time-gated Raman spectrometer was used (TimeGated® 532, TimeGate Instruments, Oulu, Finland). The system has a 532 nm picosecond pulsed laser (pulse width < 100 ps, repetition rate 40–50 kHz, average output power approximately 60 mW), sampling fiber optics, spectrometer with a complementary metal-oxide- semiconductor (CMOS) single-photon avalanche diode (SPAD) detector (8 cm^{-1} spectral resolution) and a MATLAB-based measurement software (MathWorks, Massachusetts, USA). A sampling probe (BWTek Raman Probe, B&W Tek, Newark, USA) with a focal distance of 5 mm and spot size of approximately 85 μm was used. The time-resolved spectral datasets from 0 to 2 ns were collected using the electronic delay generator (delay ramp steps 41). A window of few nanoseconds centered at around 0.5 ns was used when fluorescence subtraction was performed. For the baseline correction adaptive iteratively reweighted Penalized Least Squares (airPLS) and second-order derivatives were used.

Lastly, the polarization dependency of the Raman spectra of α-MNH was investigated using a confocal Raman microscope (Witec Alpha 300+, Germany) with a 532 nm laser and Zeiss EC Epiplan-Neofluar Dic 50× / 0.8 objective. The laser power used was 5mW (measured before the objective). Integration time was 5 seconds with 4 accumulations. The spectra of α-MNH were taken with both parallel and perpendicular polarizations by adjusting the polarization direction of the laser and analyzer of the microscope.

Complementary characterization of reference materials

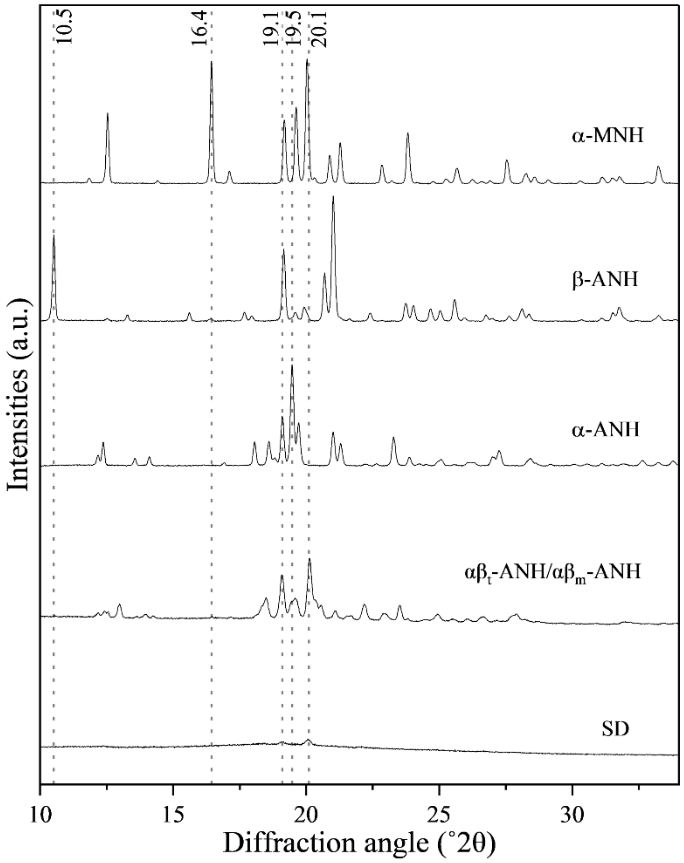

Figure S1. XRPD characterization of lactose reference materials. Some key features are marked with dashed lines on the diffractograms. The 10.5° 2θ reflection is seen in the β-ANH pattern. The 16.4° 2θ reflection, characteristic for α-MNH, suggests contamination in the β-ANH sample. The spray-dried (SD) sample exhibits a broad, nearly featureless halo, indicative of amorphousness, with minor peaks at 19.1° and 20.1° 2θ pointing to trace crystallinity associated with $\alpha\beta_t$-ANH. Additionally, the 19.5° 2θ peak is identified as a characteristic feature of α-ANH.

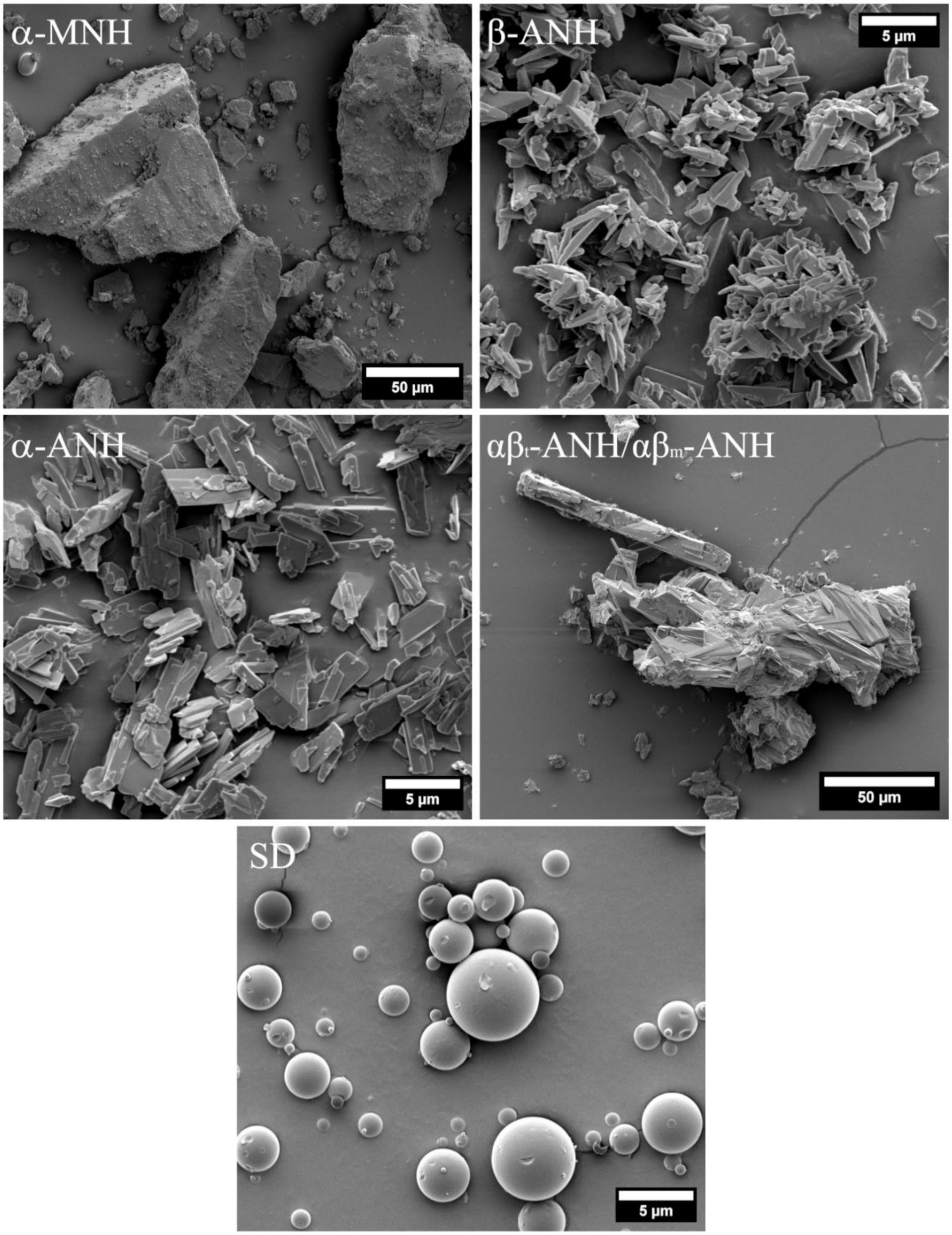

Figure S2. SEM characterization of the lactose reference materials. SEM-images of the reference materials show distinct morphological characteristics. α-MNH exhibited large crystalline structures, while α-ANH and β-ANH showed elongated, lath-like crystals with some level of polycrystallinity. The SD sample showed smooth, spherical particles typical for spray-dried materials. The $\alpha\beta_t$-ANH/$\alpha\beta_m$-ANH sample showed both columnar single crystals and polycrystalline particles.

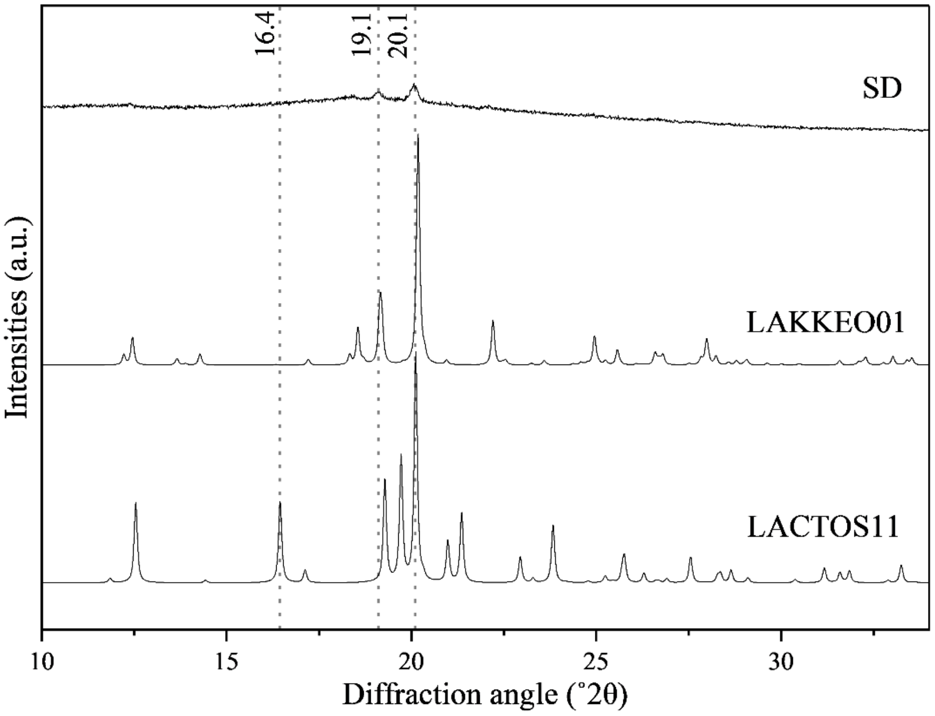

Figure S3. XRPD pattern of the SD sample showing signs of trace crystallinity and the predicted XRPD diffractograms of αβ$_t$-ANH (LAKKEO01) and α-MNH (LACTOS11). The trace crystallinity in SD lactose was identified as αβ$_t$-ANH based on peaks at 19.1° 2θ and 20.1° 2θ and the absence of the α-MNH peak at 16.4° 2θ. The slight differences in peak positions between the experimental and predicted patterns can be attributed to several factors, including instrumental factors and differences in temperature.

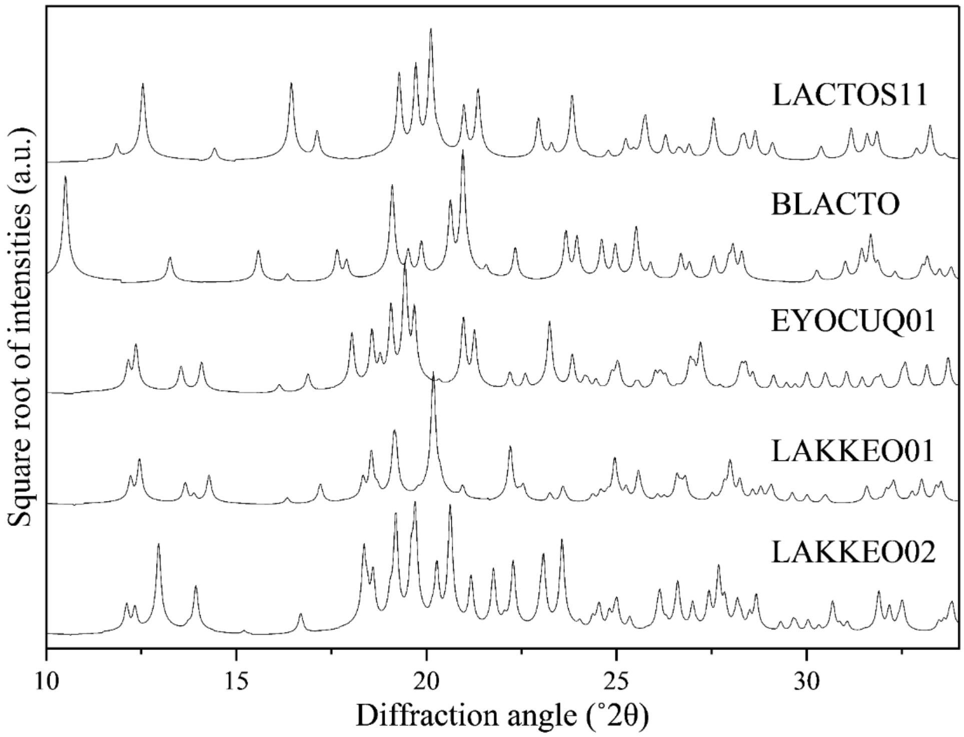

Figure S4. Predicted XRPD diffractograms from Cambridge Structural Database (CSD) structures. The patterns are labeled with their corresponding CSD refcodes: LACTOS11 (α-MNH), BLACTO (β-ANH), EYOCUQ01 (α-ANH), LAKKEO01 ($\alpha\beta_t$-ANH) and LAKKEO02 ($\alpha\beta_m$-ANH). The intensity axis has been scaled to the square root to enhance the visibility of low-intensity peaks.

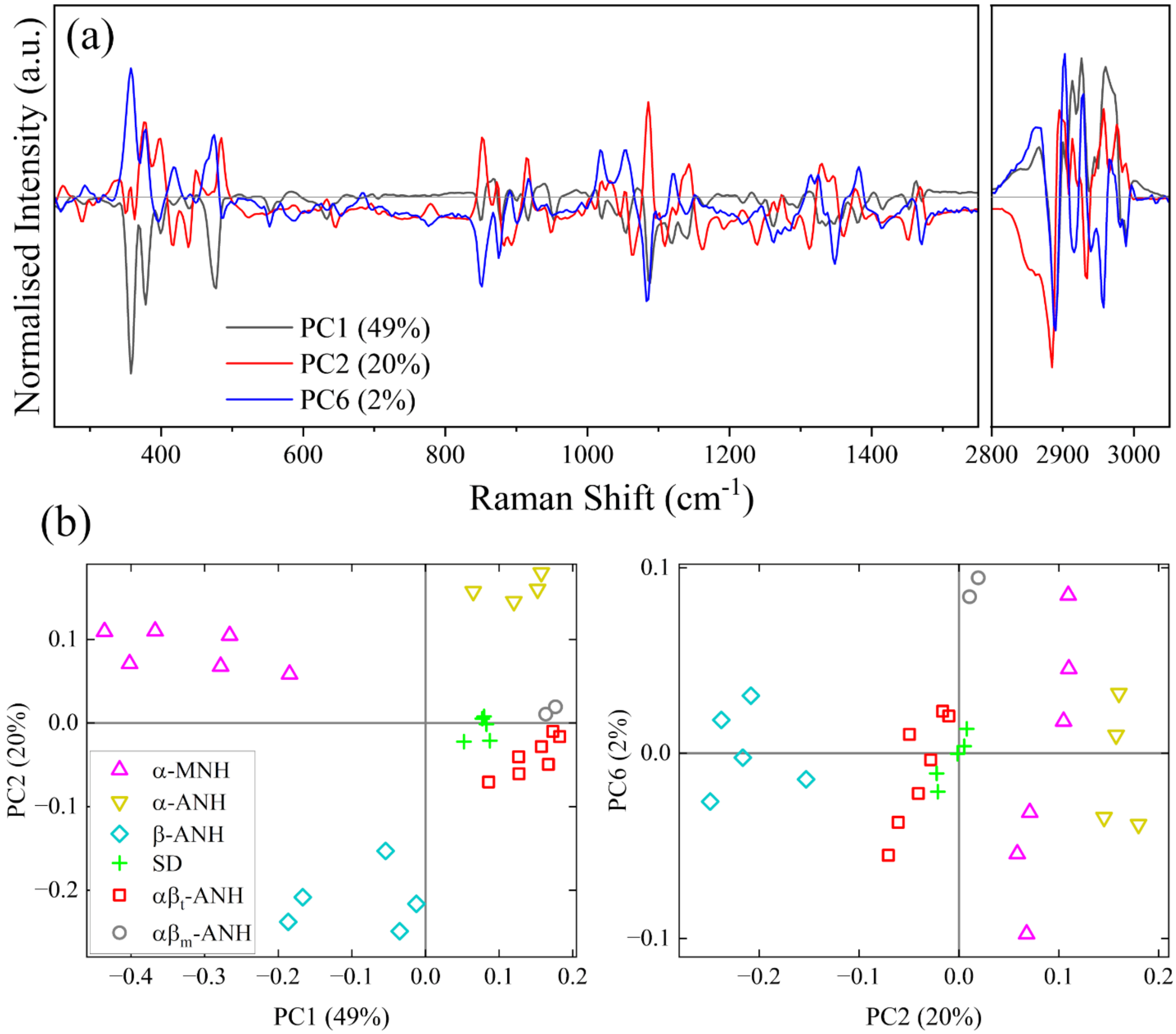

Figure S5. Principal component analysis (PCA) of spontaneous Raman microscopy spectra of the lactose reference materials. (a) The loading plots display the contributions of various spectral features to the principal components (PCs) 1, 2 and 6. (b) PCA scores plots for PCs 1, 2 and 6. The explained variances are shown in parentheses. Each symbol in the scores plot corresponds to an individual spectrum, each obtained from distinct spots within the sample. PCA effectively differentiated the lactose materials along the principal components 1, 2 and 6, as depicted in (b). The αβ$_t$-ANH/αβ$_m$-ANH mixture produced two distinct types of spectra, identified in the SRS analyses as corresponding to each of the two forms; these spectra cluster separately along PC6.

Table S2. Anomeric compositions of the reference materials, obtained with NMR

	α (%)	β (%)
**α-MNH**	98	2
**β-ANH**	8	92
**α-ANH**	97	3
**αβ$_t$-ANH/ αβ$_m$-ANH**	55	45
**SD**	47	53

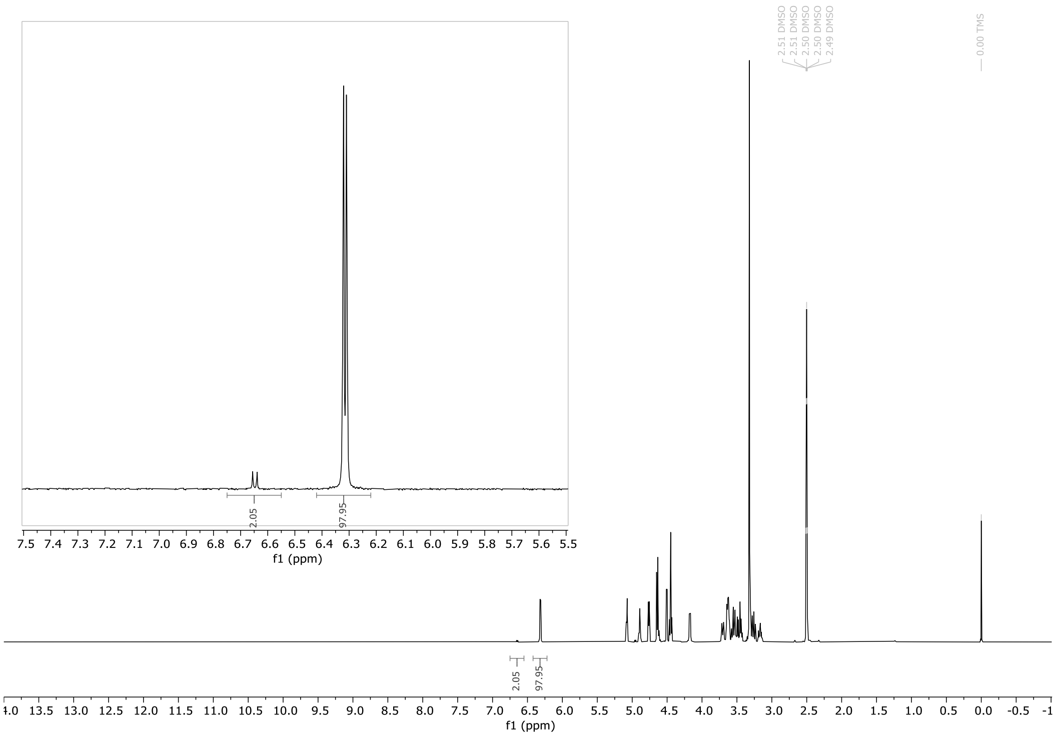

Figure S6. ^{1}H NMR spectrum of α-MNH lactose reference material

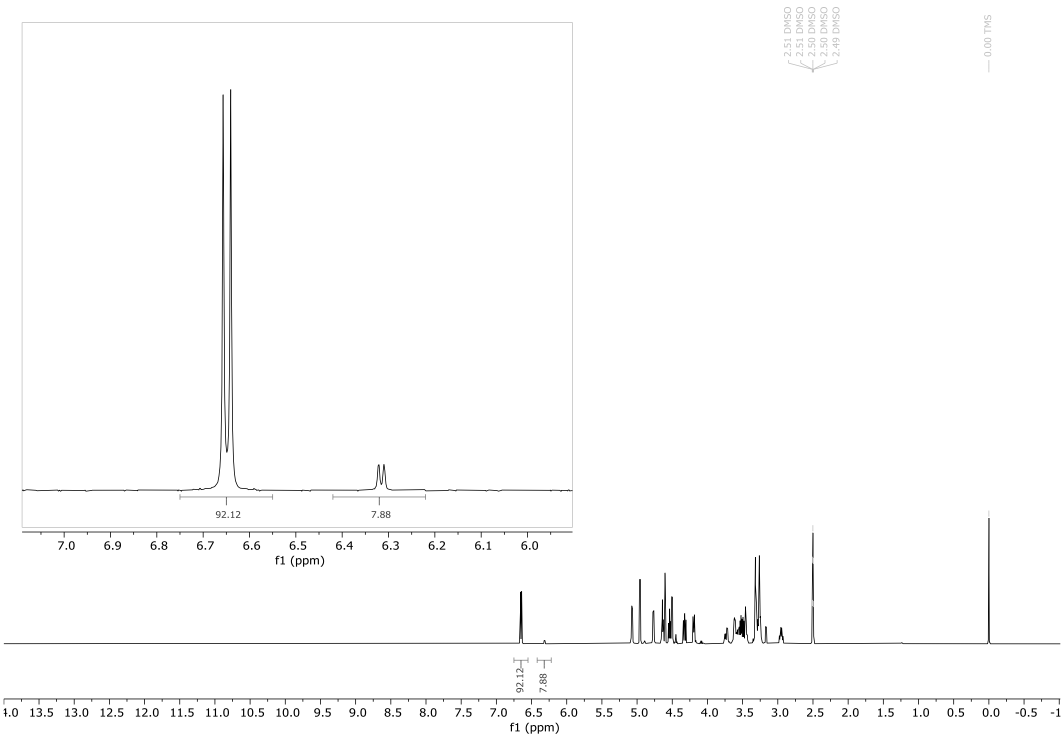

Figure S7. ^{1}H NMR spectrum of β-ANH lactose reference material

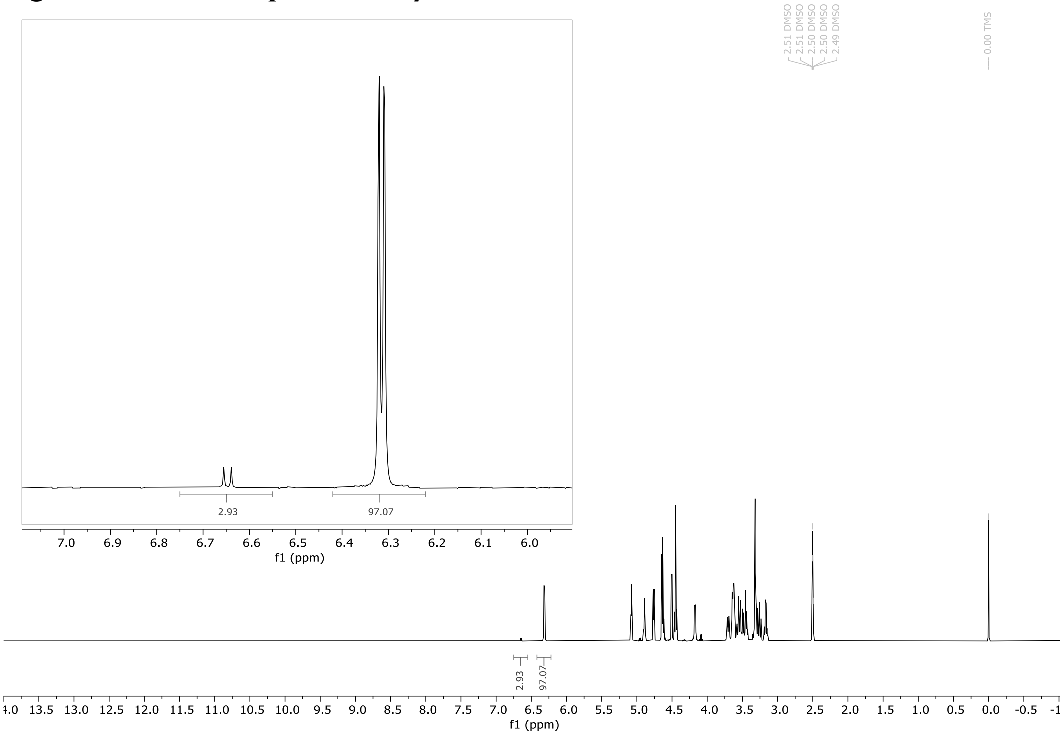

Figure S8. ^{1}H NMR spectrum of α-ANH lactose reference material

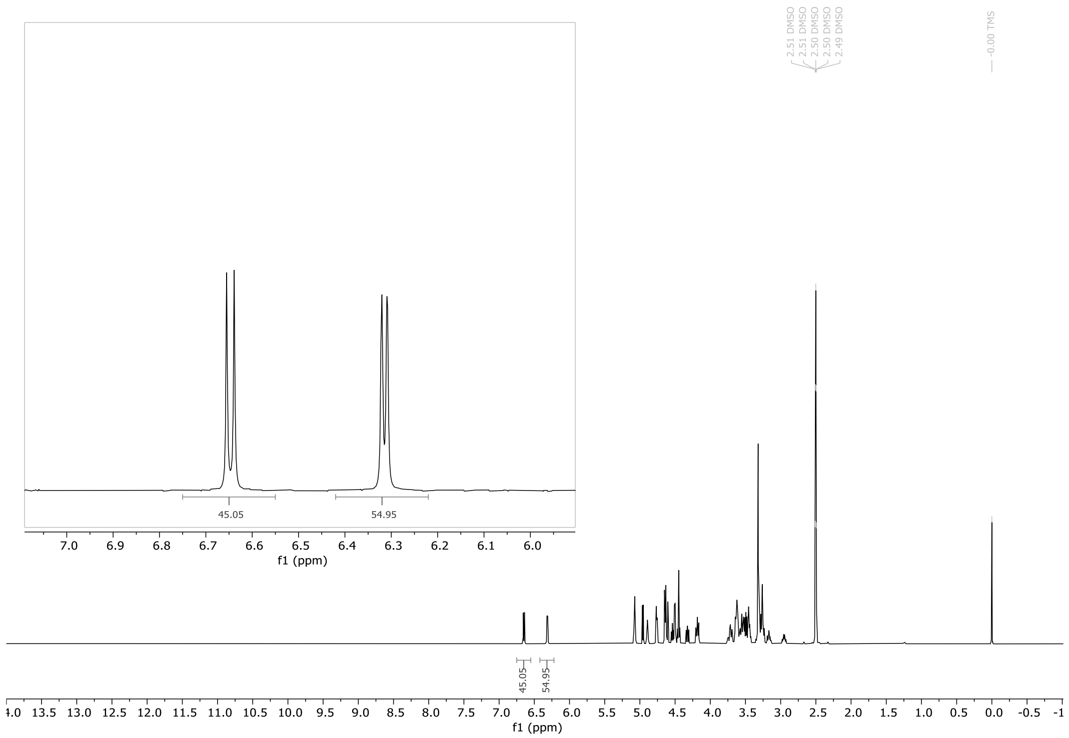

Figure S9. **^{1}H NMR spectrum of $\alpha\beta_t/\alpha\beta_t$-ANH lactose reference material**

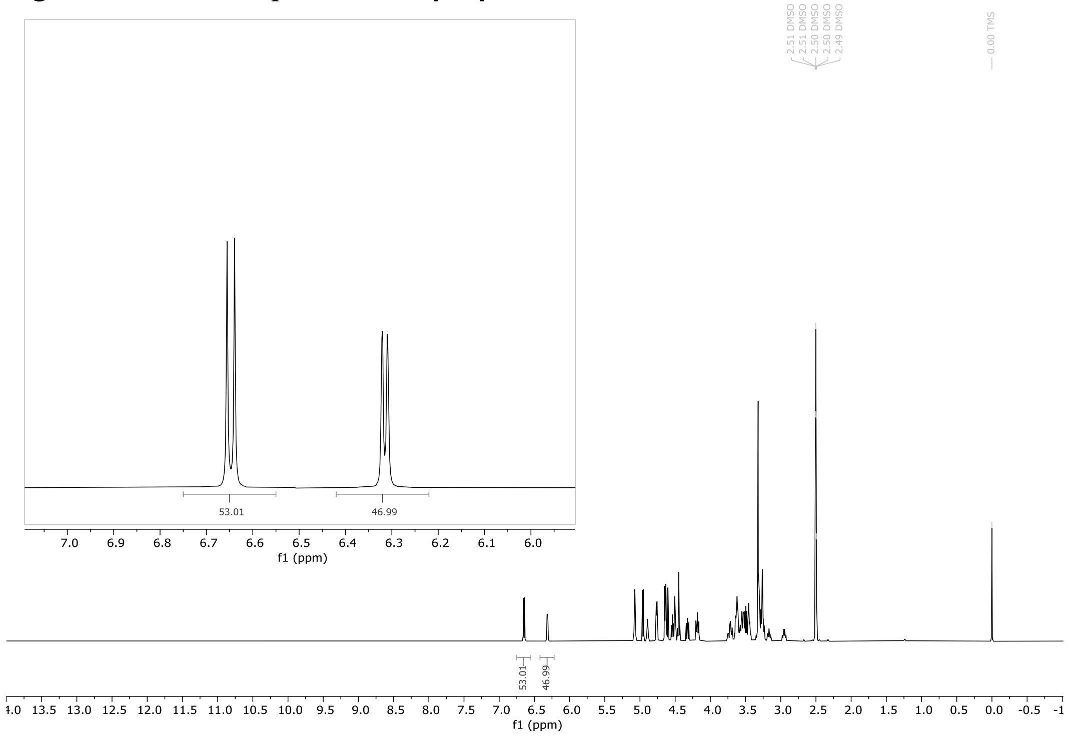

Figure S10. **^{1}H NMR spectrum of SD lactose**

Investigation of the polarization effect and its implications for data analysis

In order to assess how well two spectra can describe all orientation-dependent spectra of each crystalline form, 25 hyperspectral SRS images (141x141 μm^2 each) were measured of four raw materials: spray dried amorphous lactose (SD), alpha monohydrate (α-MNH), alpha anhydrate (α-ANH), and beta anhydrate (β-ANH). It was assumed this large set of images, each containing a high number of crystals, to contain all possible crystal orientation dependent Raman spectra, and thus studying this dataset would explain how well a set of two orientation spectra describe the data. The $\alpha\beta_t$-ANH/$\alpha\beta_m$-ANH sample was excluded from this analysis because it is a mixture of two forms.

The data analysis process was as follows. First, SRS images were pre-processed with the BM4D algorithm for noise reduction. Two orientation-dependent reference spectra (endmembers) were then extracted from the dataset with vertex component analysis (VCA). In addition, a mean spectrum of the whole dataset was calculated and compared to the set of two reference spectra. The obtained reference spectra were then used as before for CLS analysis of the dataset. From the results, the mean sum-squared signal (SSQ) captured by each component relative to the total signal in the dataset was calculated. The obtained percentages were interpreted as goodness of fit of the model, and the residual percentage describes the lack-of-fit of the model that is comparable between different choices of reference spectra. Empty regions in the images (no lactose particles) were excluded from the analysis so as to avoid background noise affecting the results. For spray-dried amorphous lactose, only the mean spectrum was used as a reference. The results are shown in **Table S3**.

The average SSQ % per component *i* is calculated as

$$SSQ\ (\%X) = \frac{C_i}{C_X} * 100\%$$

where C_i is the average sum-squared signal captured by the ith component of all pixels in the dataset, C_X is the average sum-squared signal of the whole recorded pixel spectrum such that

$$C_X = C_1 + C_2 + \cdots + C_i + C_{RES}$$

Table S3. Goodness of fit and residual analysis for different lactose forms using CLS. SSQ represents mean sum-squared signal captured by each component relative to the total signal in each dataset.

**Form**	**SSQ %**			
	**Mean spectrum**	**Reference spectrum 1**	**Reference spectrum 2**	**Residuals**
**amorphous**	96.1			3.9
**α-ANH**	93.2			6.8
		21.1	73.6	5.3
**α-MNH**	92.2			7.8
		18.5	76.7	4.8
**β-ANH**	93.7			6.3
		86.9	7.3	5.8

Spray-dried amorphous lactose has no crystalline structure and thus, should not have any orientation or polarization dependent differences in its Raman spectra. Therefore, it was used as a reference to compare other results to, as its residual SSQ describes the experimental error and noise present in the measurements. The results show that even if the crystalline lactose forms are described only with one mean spectrum, the residual SSQ is only slightly higher than from the orientation-dependent reference spectra case. As seen in **Table S3** quite reliable identification of the lactose forms is possible with only one reference spectrum per lactose form. As expected, increasing the number of reference spectra in the CLS model lowers the residuals. Overall, the lack-of-fit caused by approximative description of the polarization effects was interpreted to be insignificant in the context of the study's qualitative and quantitative analyses of the lactose samples.

The spectra extracted with VCA (extended approach) are shown in **Figure S11**, together with the spectra obtained using the method described in the article (faster approach). The comparison shows that the extended approach and the method used in the article yield very similar spectra.

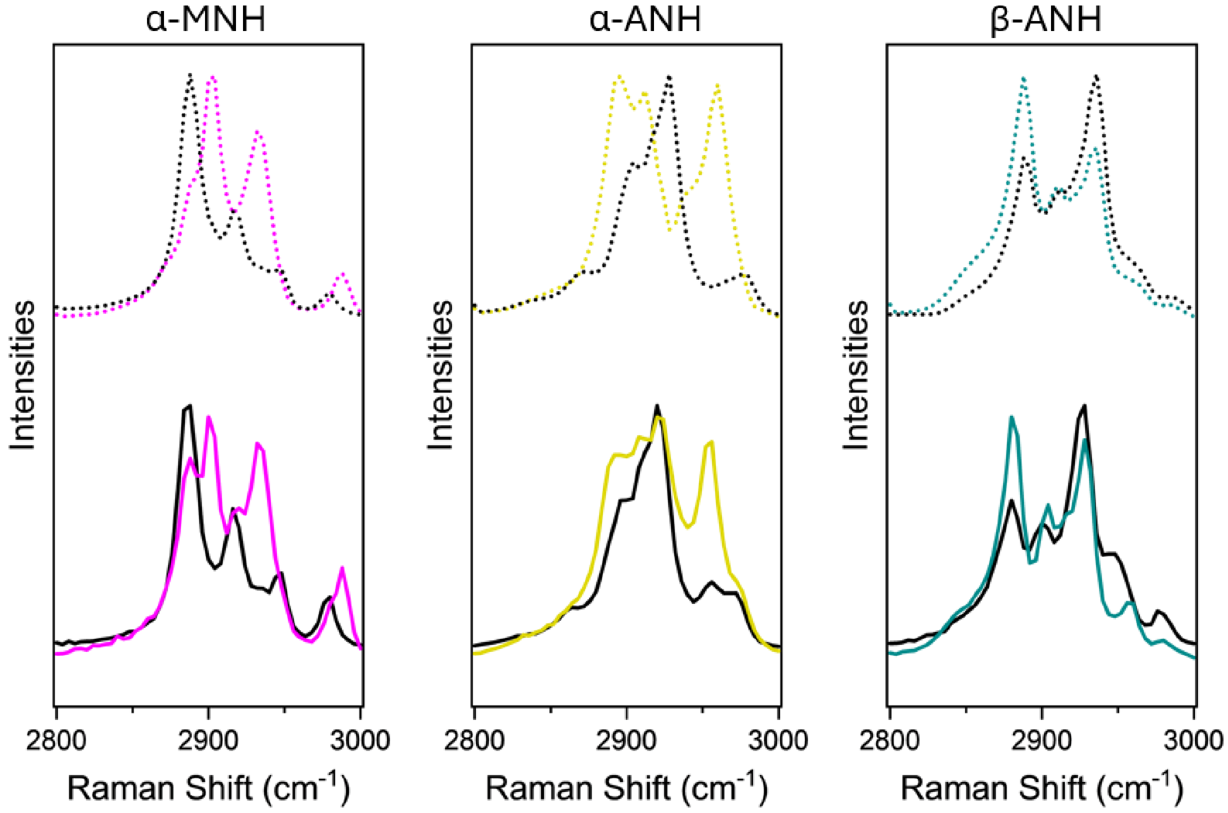

Figure S11. Spectra obtained with the faster (solid lines) and extended approach (dashed lines). The spectra obtained with the faster approach were selected from f-SRS images taken under P and S polarizations from particles which showed clear spectral differences between the two polarization states. The spectra obtained with the extended approach were extracted from a large number of epi-SRS images of each form, by using vertex component analysis.

To further demonstrate the robustness of using two input spectra for each crystalline compound, three single-pixel spectra from an image of a sample containing α-MNH, α-ANH and β-ANH were randomly selected. In **Figure S12**, both the observed and modeled spectra are presented, along with the residual spectra for these pixels. In all three pixels, almost all of the spectral features were accurately accounted for by the two input spectra specific to

each component. The minor contributions from other components (**Figure S12 (e)**) in the α-MNH pixel are believed to be instances of overfitting.

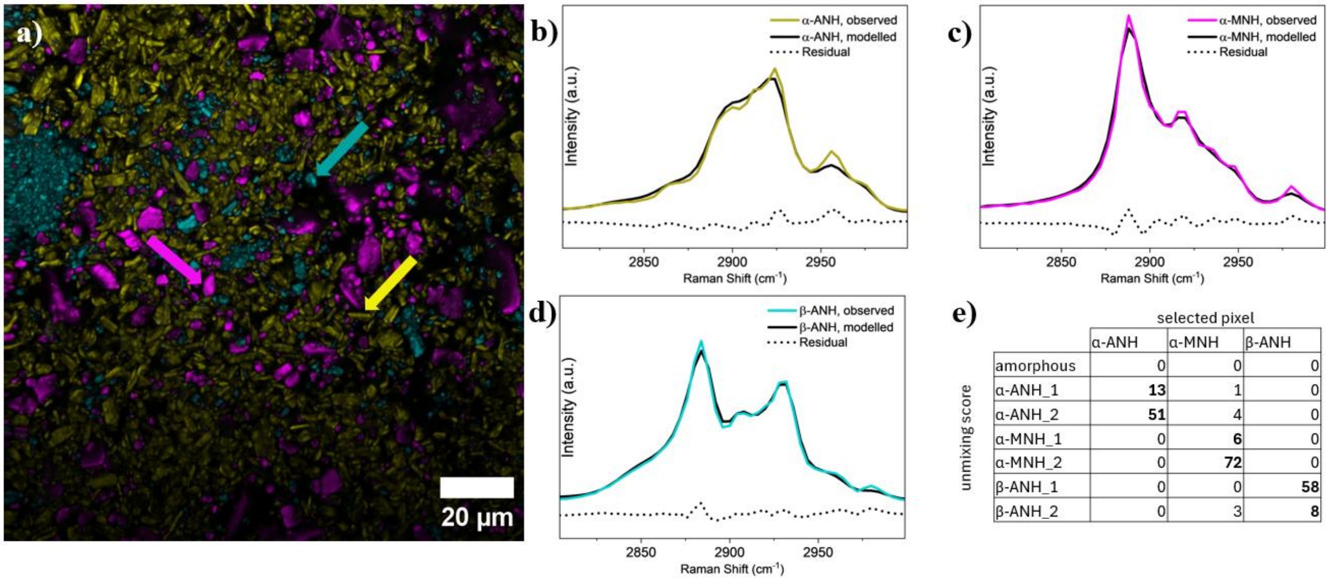

	α-ANH	α-MNH	β-ANH
amorphous	0	0	0
α-ANH_1	**13**	1	0
α-ANH_2	**51**	4	0
α-MNH_1	0	**6**	0
α-MNH_2	0	**72**	0
β-ANH_1	0	0	**58**
β-ANH_2	0	3	**8**

Figure S12. (a) SRS image of the lactose mixture containing β-ANH, α-ANH and α-MNH lactose. The arrows indicate pixels where the spectra in (b–d) were extracted. (e) Raw score values for each fitted component at the selected pixels, with values in bold indicating the correct contribution

The usage of circularly polarized light in forward-detected direction (f-SRS) (**Figure S13**), which could potentially reduce the orientation effects, was also explored. The downside of f-SRS is that its application is limited to the analysis of sufficiently transparent samples, i.e. a thin layer of powder samples. This restriction poses a significant limitation in pharmaceutical applications, particularly when imaging the surfaces of tablets. The results (**Figure S13**) indicated that, compared to epi-SRS, circular polarization in f-SRS was not particularly effective in reducing the polarization-dependent variations in the signal.

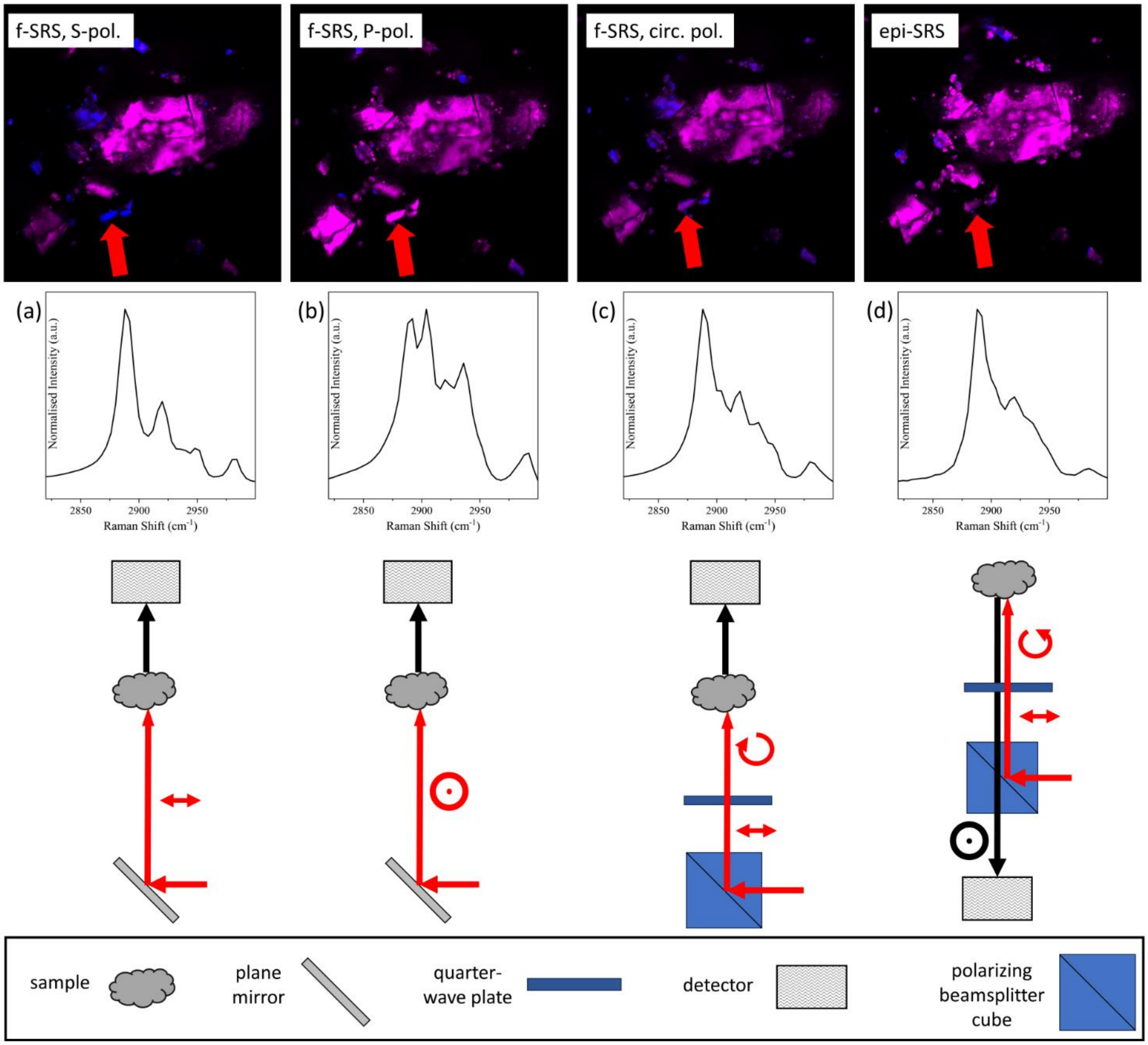

Figure S13. SRS images of α-MNH (from the same sample area), measured using either linearly or circularly polarized light in the forward or backward scattered direction. Spectra (a-d) were plotted from the particle indicated by the red arrows. The false-colored images were generated with CLS, using the spectra a and b as inputs. The images are 141x141 μm^2 in size. Below are schematic illustrations corresponding to each of the four polarization configurations. Epi-SRS configuration was used in the characterization of all the samples in the study.

In the unmixing coefficient-based quantification of different lactose solid-state forms, the input spectra were scaled based on their approximated Raman cross-sections. For this purpose, all reference materials were measured with a time-gated Raman instrument (**Figure S14**). To account for differences in optics between the two polarizations, the P-polarized spectra were downscaled by a factor of 0.87, derived from the amorphous lactose spectra obtained under both polarizations. The focusing conditions were estimated to be identical for both polarization orientations.

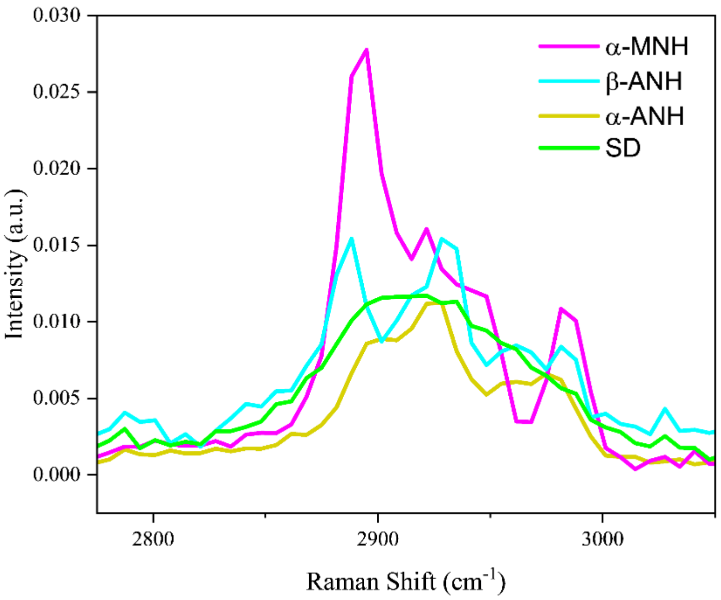

Figure S14. Raman spectra of the lactose reference materials, measured with the time-gated Raman instrument and shown as averages of three measurements. The relative intensities of these spectra were used to approximate the Raman cross-section of different lactose solid-state forms.

NMR spectra of the commercial samples

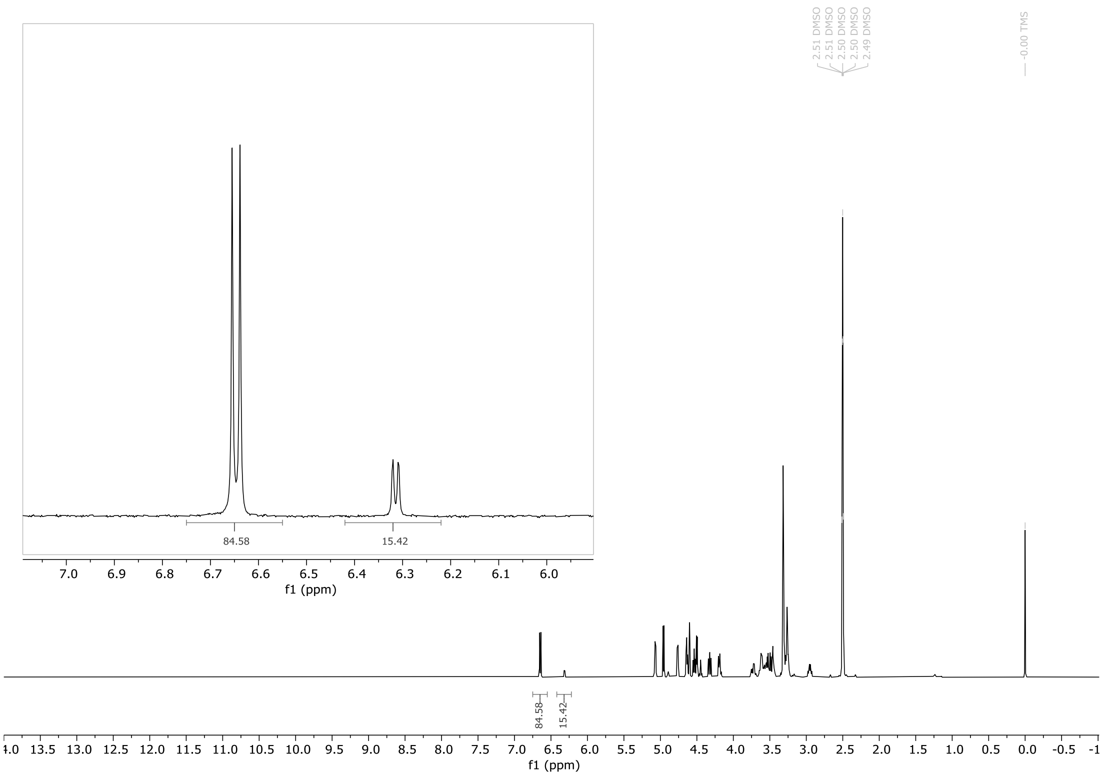

Figure S15. ^{1}H NMR spectrum of Lactohale® 400

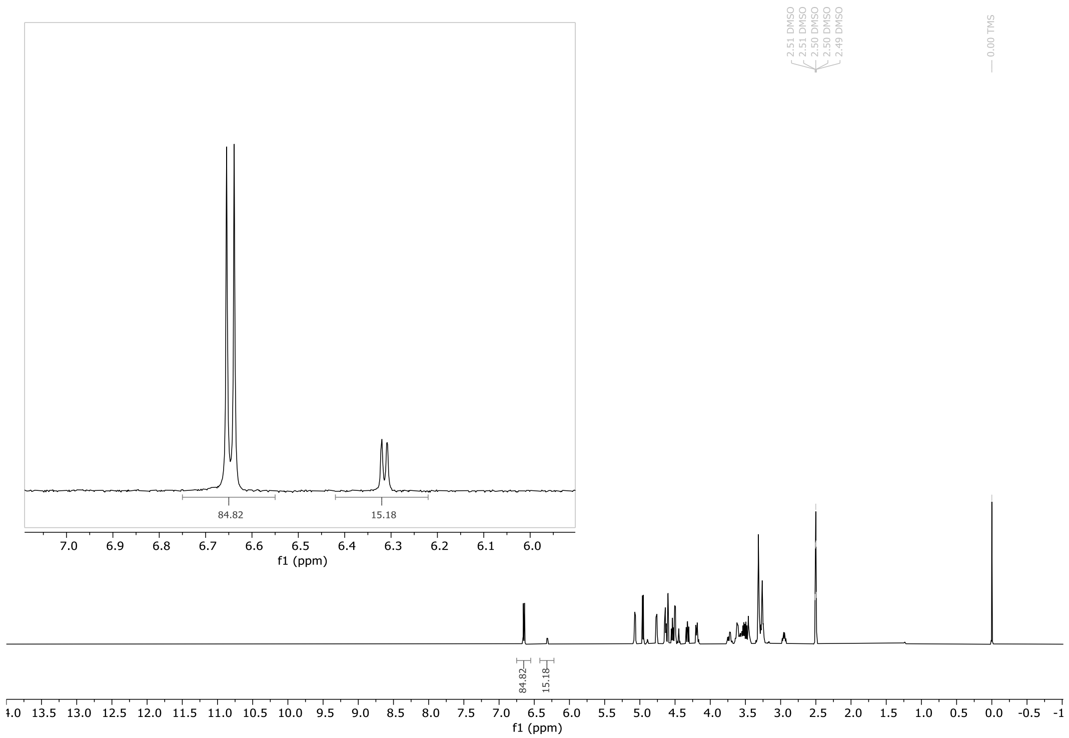

Figure S16. ^{1}H NMR spectrum of conditioned Lactohale® 400

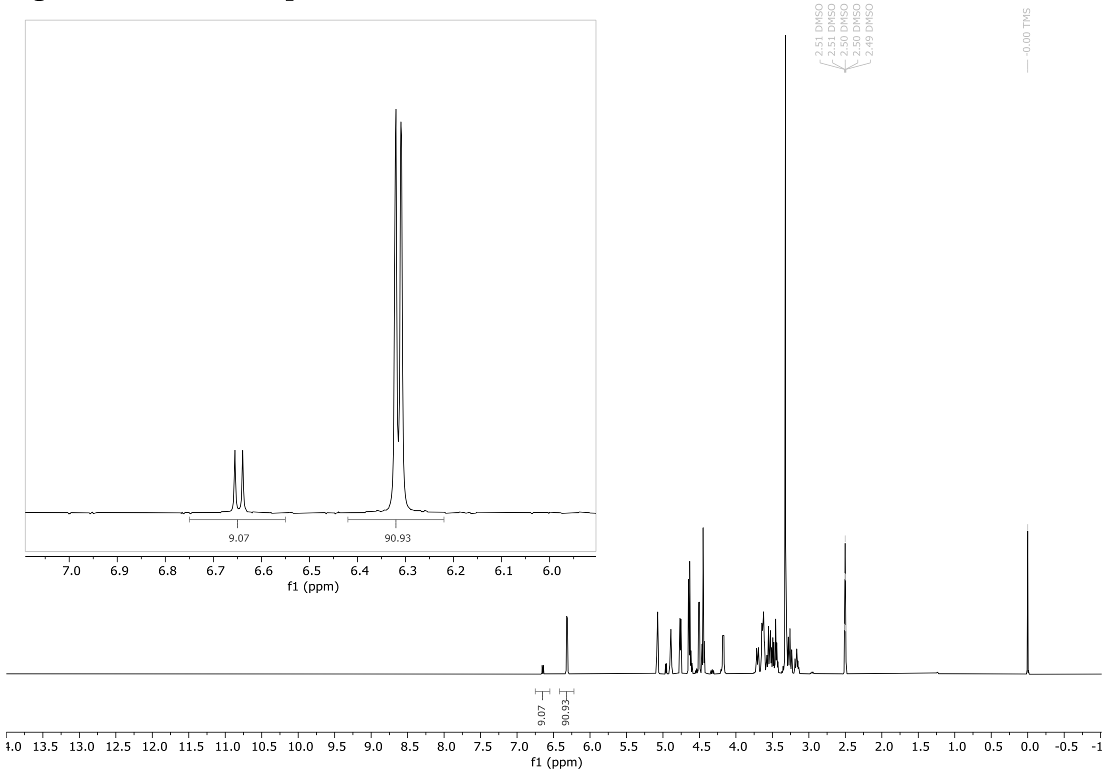

Figure S17. ^{1}H NMR spectrum of Lactopress® Granulated

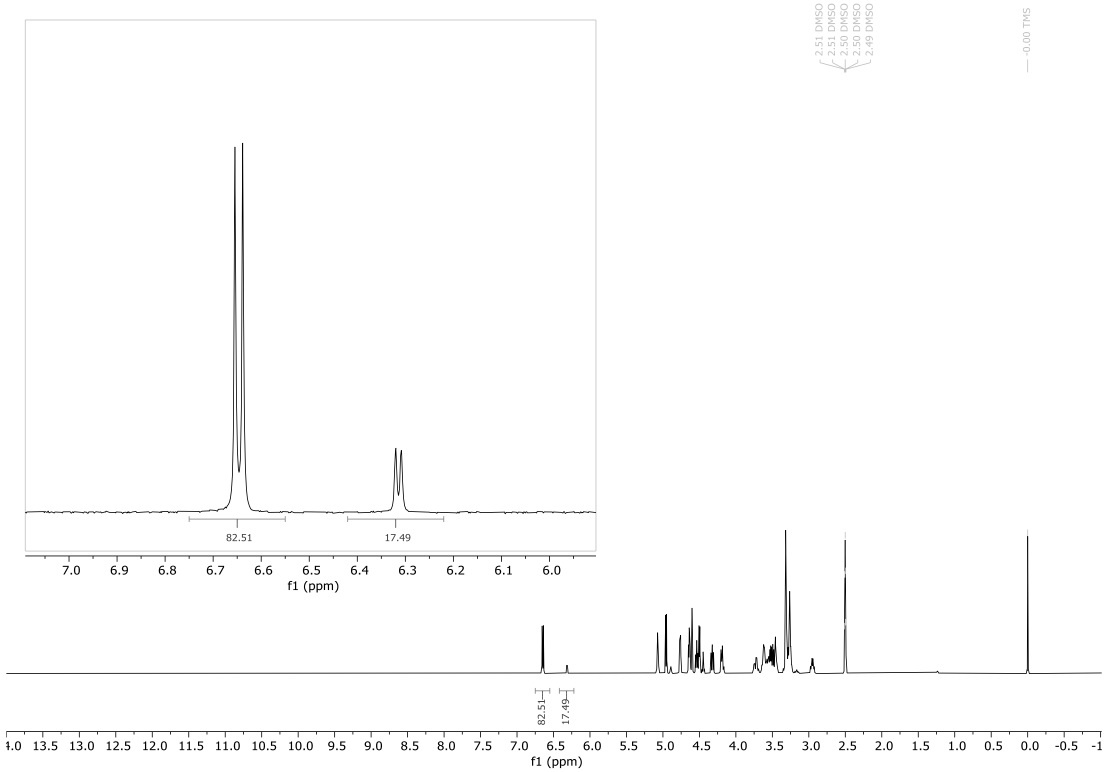

Figure S18. ^{1}H NMR spectrum of SuperTab® 24AN

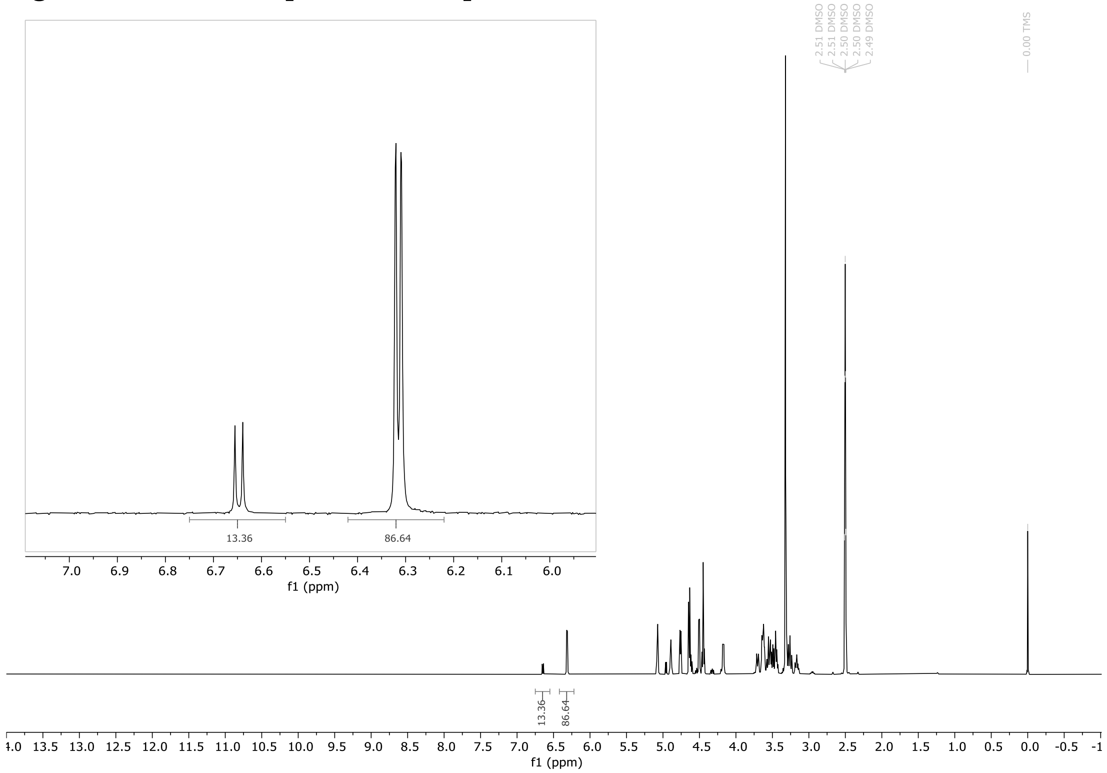

Figure S19. ^{1}H NMR spectrum of SuperTab® 14SD

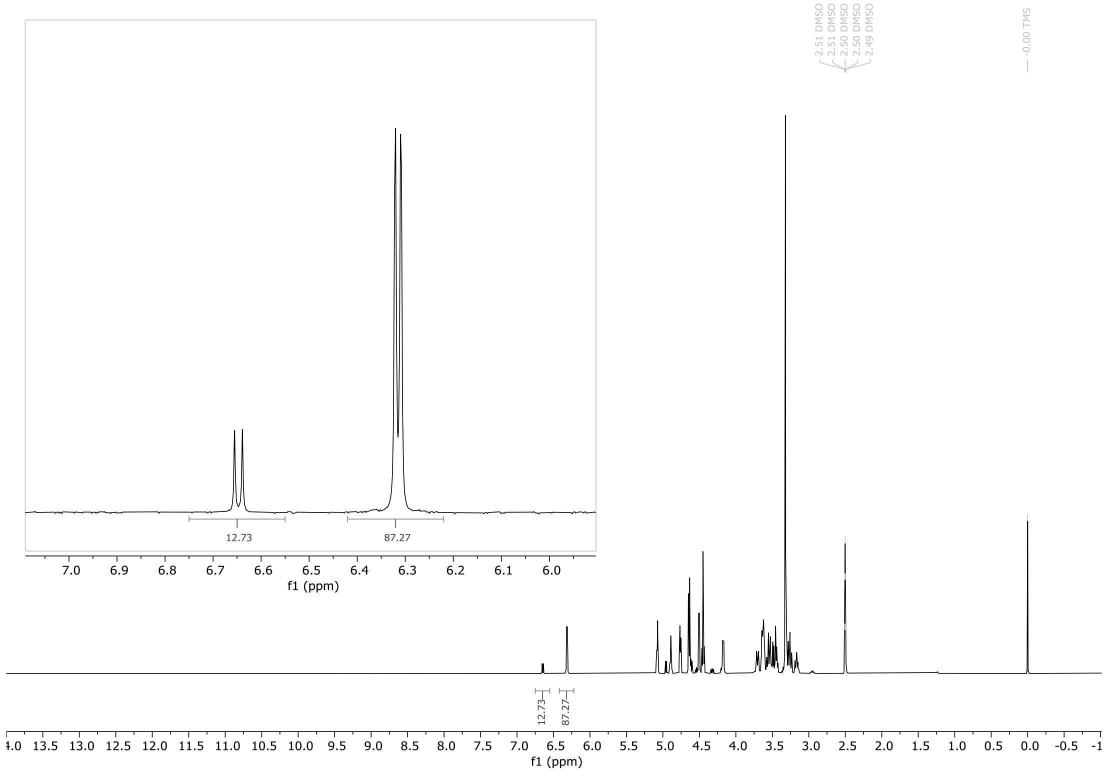

Figure S20. ^{1}H NMR spectrum of conditioned SuperTab® 14SD

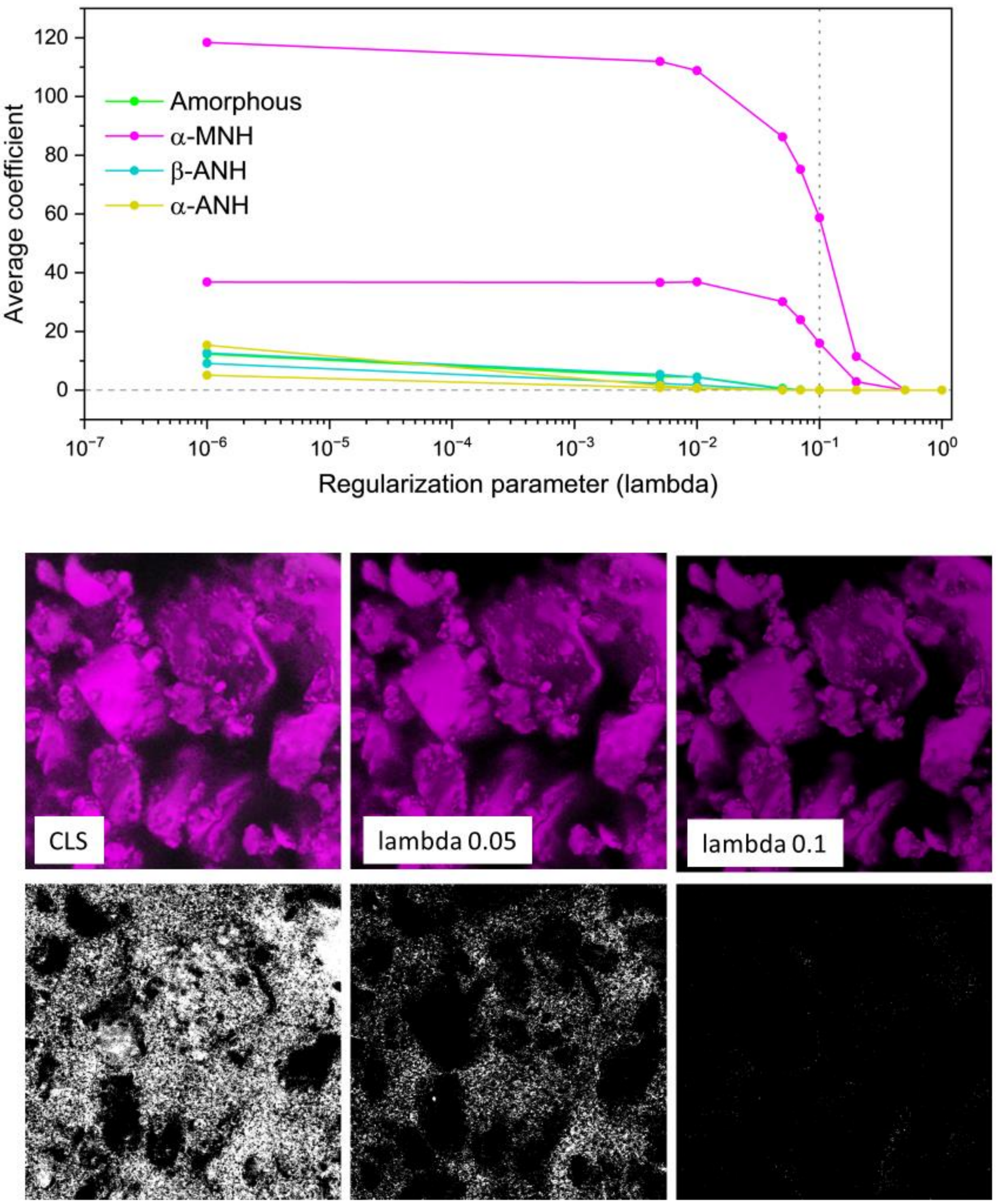

Figure S21. Above: Trace plot displaying the average coefficient for each component (two input components for each crystalline form) at varying degrees of regularization. Without regularization (or with minimal regularization), all input components are modeled as being present, regardless of their actual presence in the sample. As regularization is increased, the overfitting decreases; the amorphous signal overlapping with α-MNH particles disappears first, and as the regularization is further increased also the overfitted signal from the cover glass background disappears. The dashed vertical line indicates the lambda value of 0.1, selected for the analyses. Below: False-colored images (sized 141x141 μm^2) generated using (non-regularized) CLS and regularized LASSO at lambda values of 0.05 and 0.1. Beneath each image, a binary image is shown where white represents non-zero pixels in the amorphous channel.

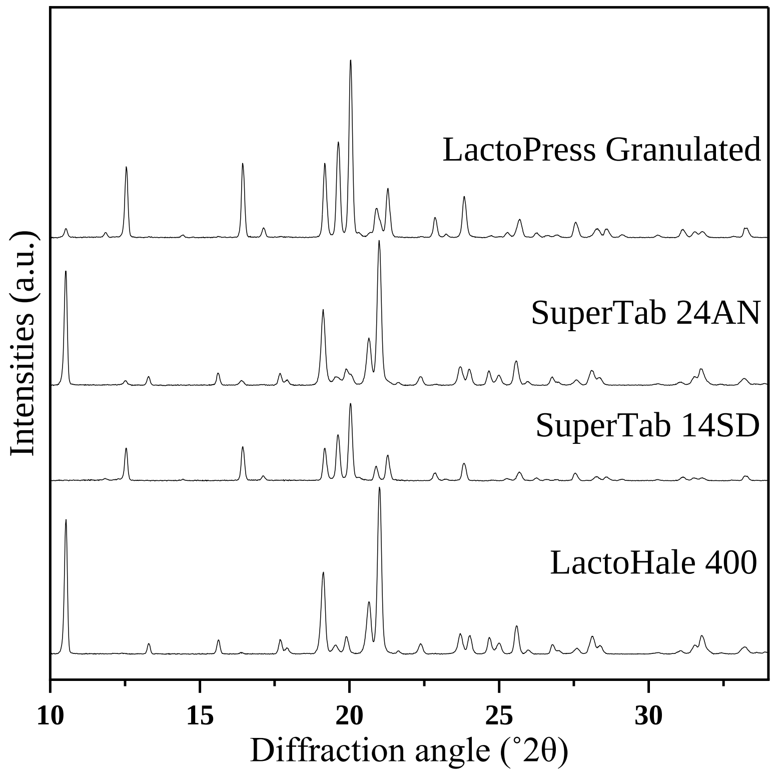

Figure S22. XRPD diffractograms of the commercial lactose samples

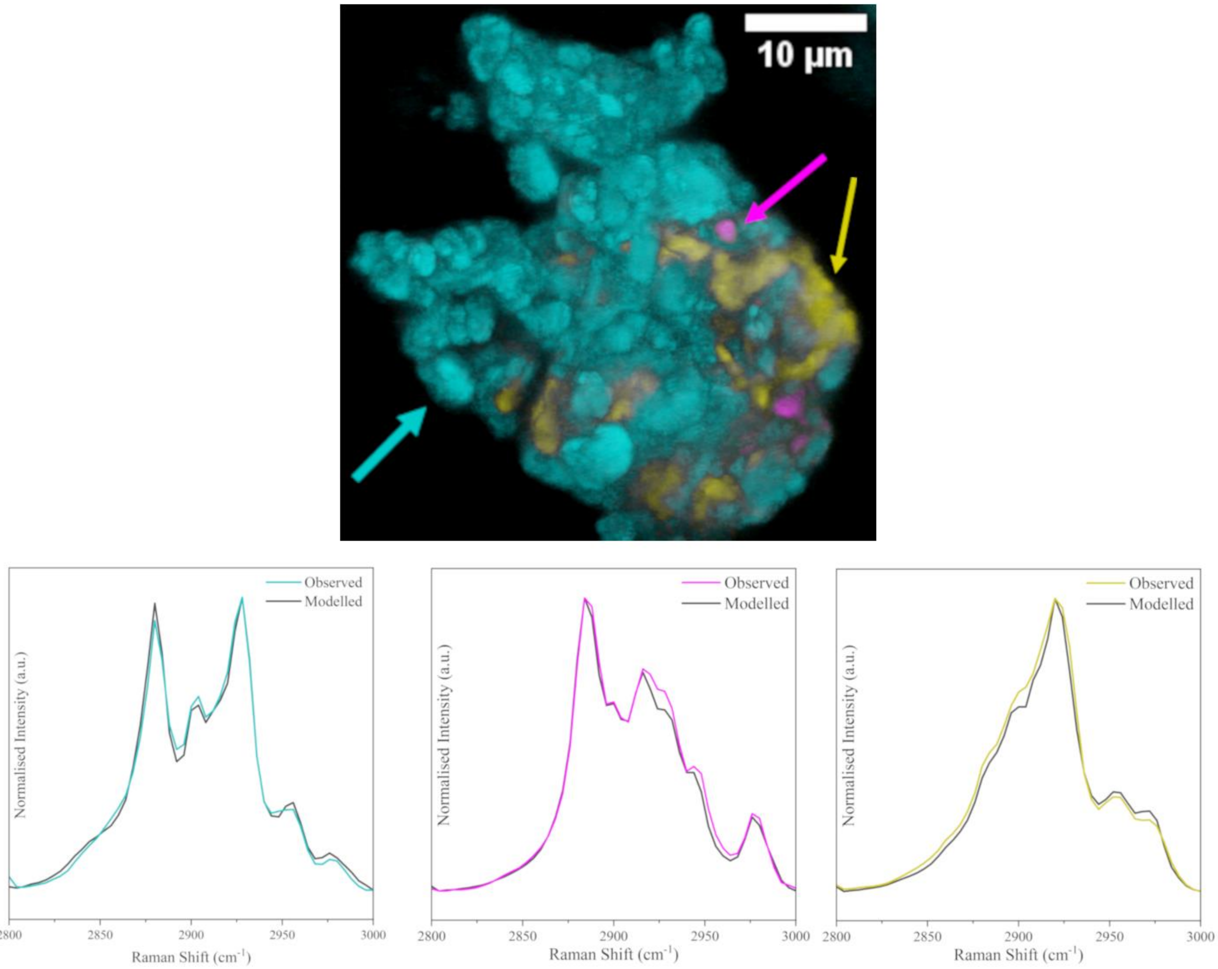

Figure S23. SRS characterization of Lactohale® 400. The SRS images are maximum Z-projection overlays, where each color-coded channel indicates a different solid-state form of lactose: β-ANH (cyan), α-MNH (magenta) and α-ANH (yellow). Arrows on the SRS image indicate the regions where the observed and modelled spectra were plotted.

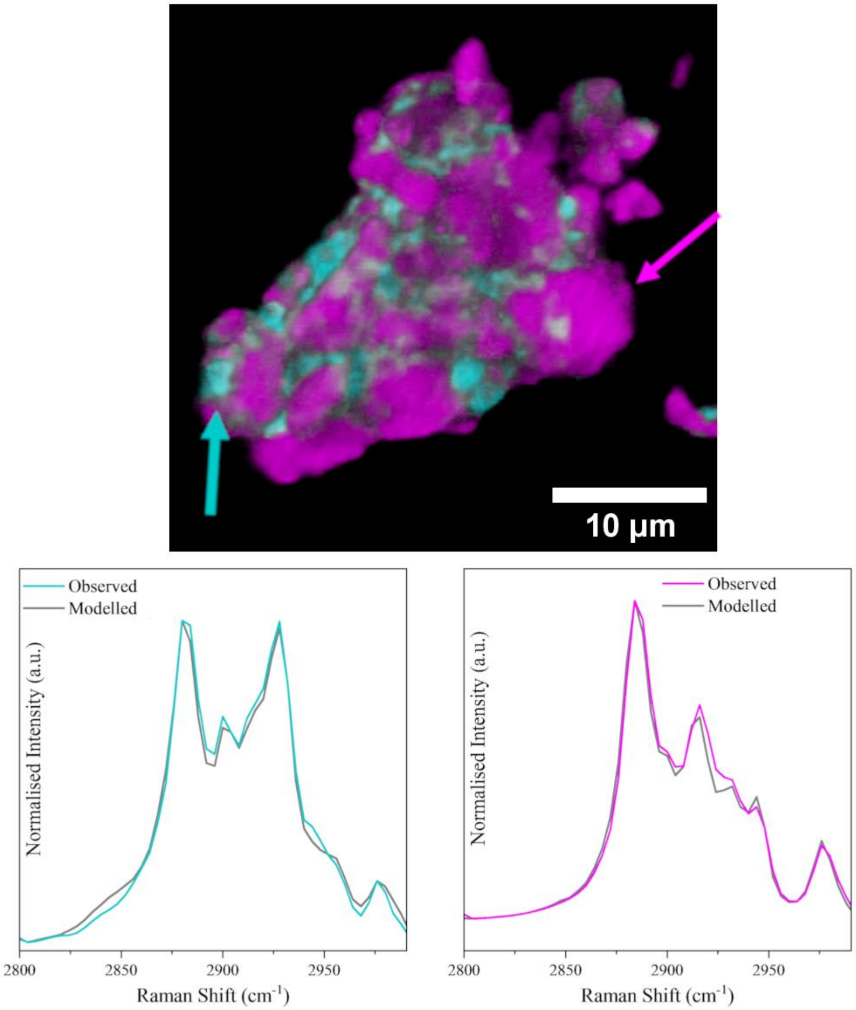

Figure S24. SRS characterization of Lactopress® Granulated. The cyan and magenta colors represent β-ANH and α-MNH, respectively. Arrows on the SRS image indicate the regions where the observed and modelled spectra were plotted.

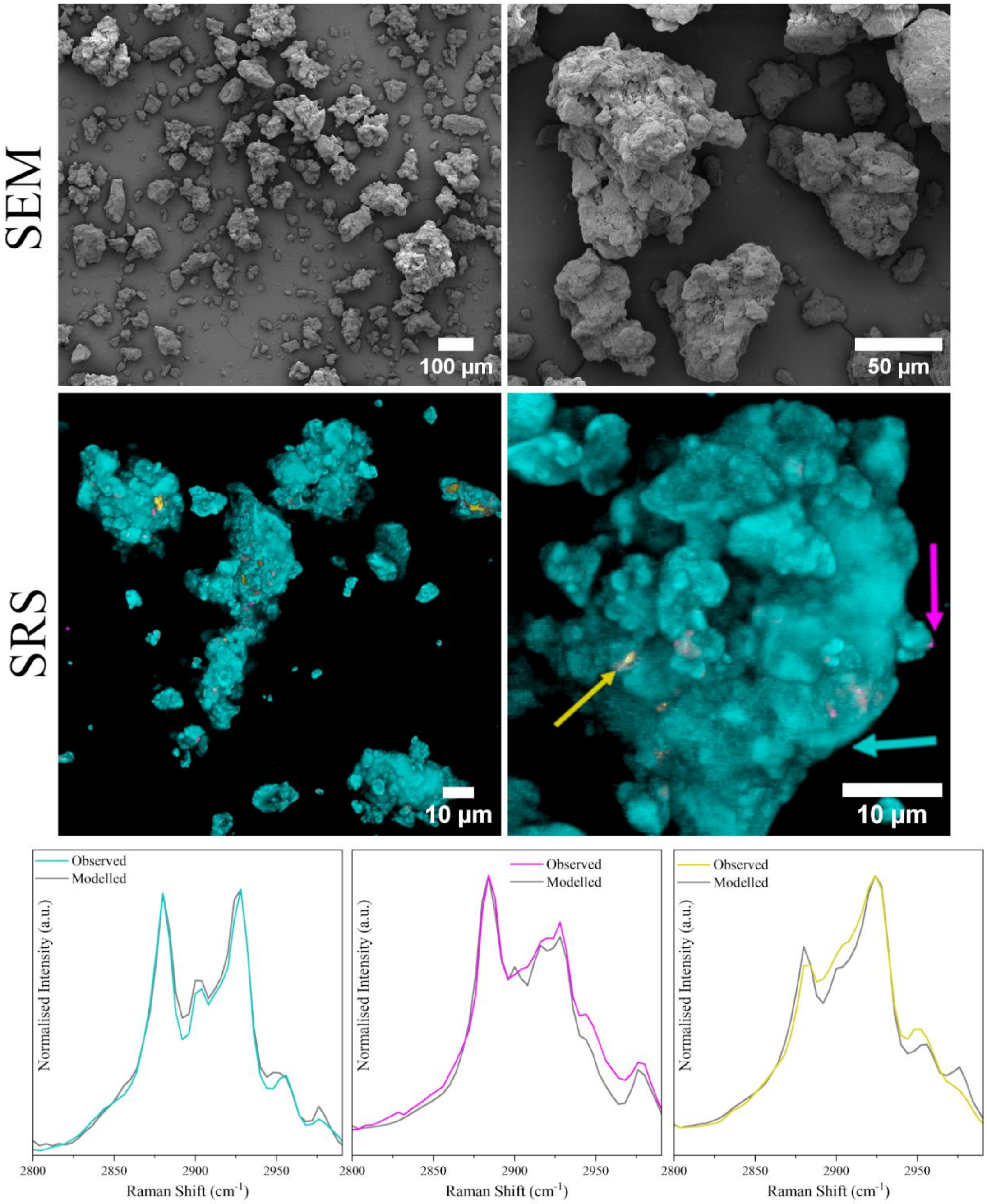

Figure S25. SEM and SRS characterization of SuperTab® 24AN. It primarily contains β-ANH, with traces of α-ANH and α-MNH forms. The SRS images are maximum Z-projection overlays, where each color-coded channel indicates a different solid-state form of lactose: β-ANH (cyan), α-MNH (magenta) and α-ANH (yellow). The trace amounts of the minor forms were predominantly blended with β-ANH at sub-resolution level but, were detectable from mixed pixels with the help of LASSO unmixing. Arrows on the SRS image indicate the regions where the observed and modelled spectra were plotted.

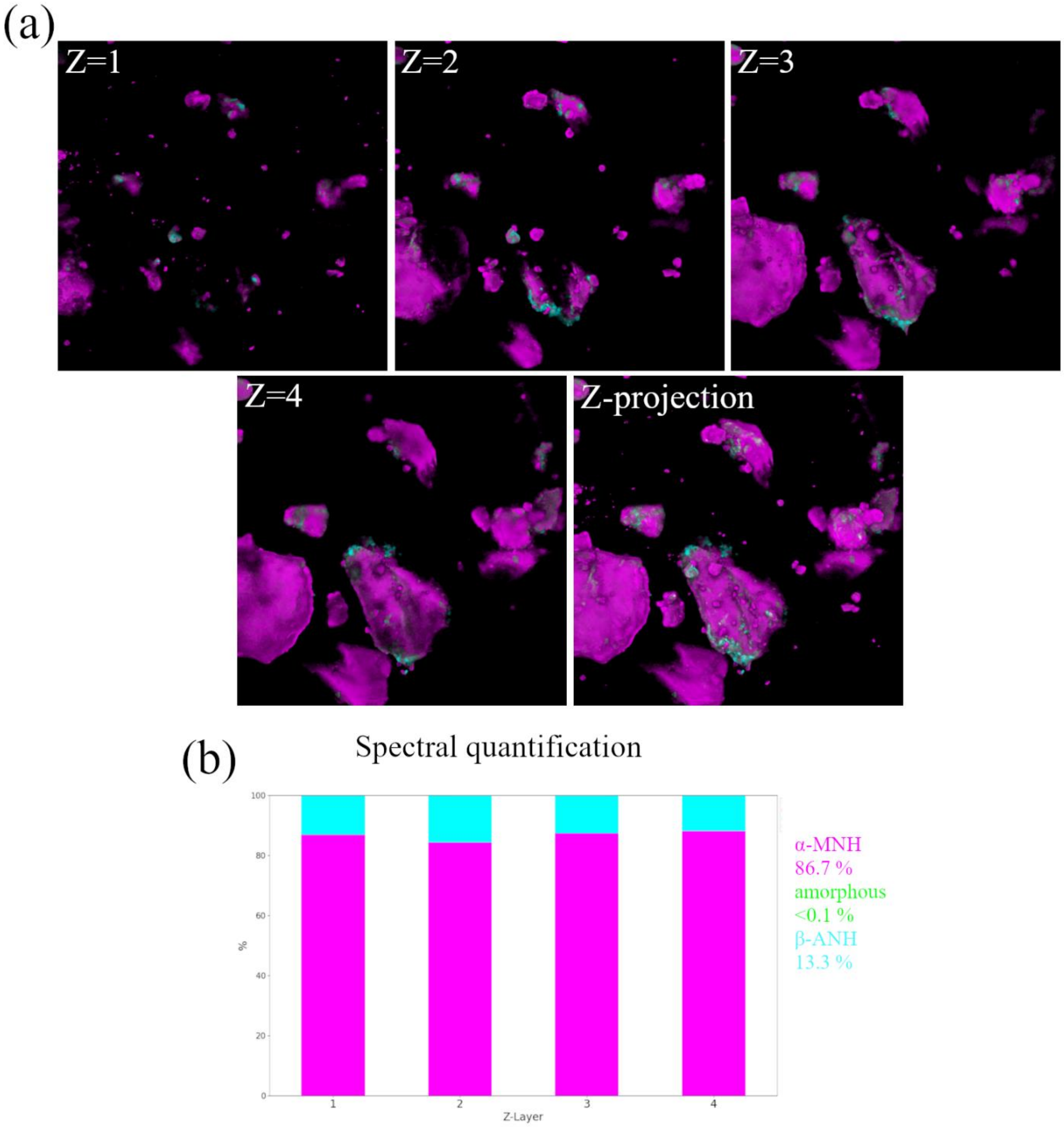

Figure S26. Example of the quantification of one SRS Z-stack of Lactopress® Granulated sample. (a) SRS images from Z-levels 1–5 and the maximum Z-projection image of those. The cyan and magenta colors represent β-ANH and α-MNH, respectively. (b) Spectral quantification based on unmixing coefficients, calculated for each Z-layer. The values on the right side show averages of all Z-layers of the Z-stack.

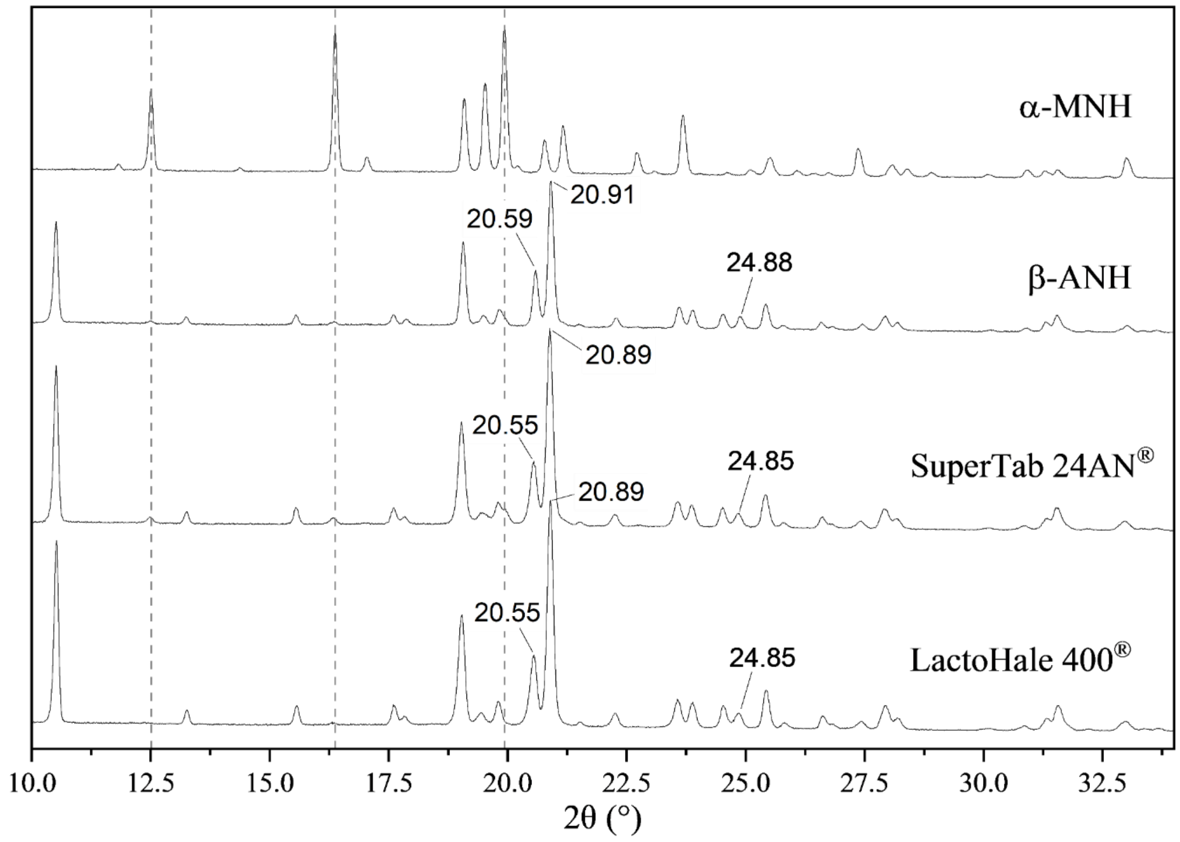

Figure S27. XRPD patterns of α-MNH, β-ANH, Lactohale® 400 and SuperTab® 24AN. Dashed grey lines indicate peak positions characteristic to α-MNH, observed as an impurity in β-ANH and possibly in Lactohale® 400 through a very low intensity peak. Clearer traces of α-MNH were detected in SuperTab® 24AN. The positions of the β-ANH peaks where slight differences were observed between β-ANH and the commercial samples are marked.

Quantitative analysis

Calibration measurements

Calibration measurements were conducted to assess the method's quantification capabilities. Additionally, different quantification approaches were compared (non-regularized CLS, regularized LASSO and area-based quantification). First, fresh batches of the four reference materials were prepared (amorphous spray-dried lactose, α-MNH, α-ANH and β-ANH). These materials were confirmed to contain no other solid-state forms of lactose as contaminants, according to XRPD, SRS, and SFG. While the α-ANH sample did contain a trace of an unidentified contaminant, as detected with SRS (**Figure S33**), it was considered insignificant in the context of these analyses. Eleven mixtures (300 mg of each) with varying concentrations were then prepared (as detailed in **Table S4**) and quantitatively analyzed these mixtures with SRS, XRPD and solution-based NMR. In the experimental design, a quaternary mixture experimental design was followed, and **Table S4** presents the actual weighed concentrations.

Preparing homogeneous mixtures of solid powders is not a trivial task, as variations in particle size and morphology can lead to segregation. To minimize segregation, α-MNH was ground and passed through a sieve with a mesh size of 71 µm. To recrystallize any potential amorphous content (although considered unlikely) created during grinding, the sample was kept at 56% RH for two days following the grinding. To maximize homogeneity, all components were added sequentially in descending order of concentration (m/m%) to a mortar and each component was thoroughly mixed with spatula before the addition of the next component. Visible lumps were broken down with a pestle, but grinding was avoided to prevent inducing solid-state transformations.

Table S4. Mixture samples prepared from amorphous spray-dried lactose, α-MNH, α-ANH and β-ANH (m/m%)

label	beta ANH %	alpha ANH %	alpha MNH %	Amorphous %
1	60.8	12.6	13.3	13.3
2	13.9	59.1	13.9	13.2
3	14.7	15.4	53.7	16.1
4	15.1	12.5	14.1	58.2
5	26.1	24.8	23.0	26.1
6	25.3	26.0	22.8	26.0
7	24.3	26.3	25.0	24.3
8	34.2	31.2	34.6	0.0
9	0.0	32.5	33.2	34.3
10	32.6	0.0	33.6	33.9
11	33.8	31.5	0.0	34.7

The three crystalline forms in the mixtures were quantified using XRPD by measuring the intensities of specific diffraction peaks (after baseline-correction), at 10.55° 2θ for β-ANH, 16.5° 2θ for α-MNH, and 18.64° 2θ for α-ANH (**Figure S28**). The peaks were chosen for their high intensity and minimal overlap between the different forms. The calibration curves show R^2 values between 0.94–0.97.

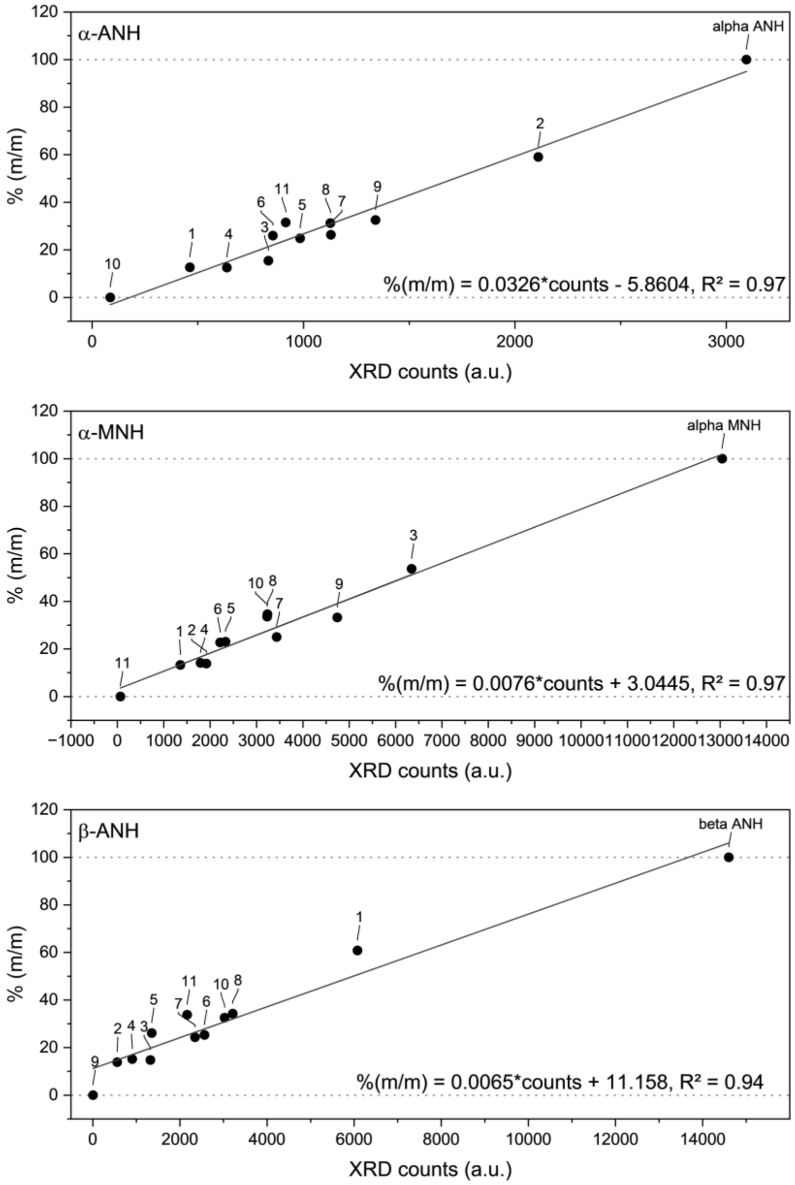

Figure S28. XRPD standard curves for α-ANH, α-MNH and β-ANH, constructed using the mixture samples.

Anomeric compositions of the 11 mixture samples and pure reference materials were analyzed with solution-based NMR (**Table S5**). Based on the measured anomeric compositions of the pure reference materials, the expected anomeric compositions in the 11 mixture samples were calculated and compared with the measured values (**Figure S29**). It should be noted that less than 10% of each bulk sample (300 mg) was analyzed in the NMR experiments, so the unrepresentativeness of these subsamples was a likely source of error in this analysis.

Table S5. Anomeric compositions of the mixture samples and pure reference materials, obtained with NMR spectroscopy.

sample	alpha anomer (%)	beta anomer (%)
1	68	32
2	24	76
3	25	75
4	50	50
5	42	58
6	43	57
7	41	59
8	35	65
9	21	79
10	51	49
11	55	45
alpha ANH	97	3
alpha MNH	97	3
beta ANH	5	95
amorphous	40	60

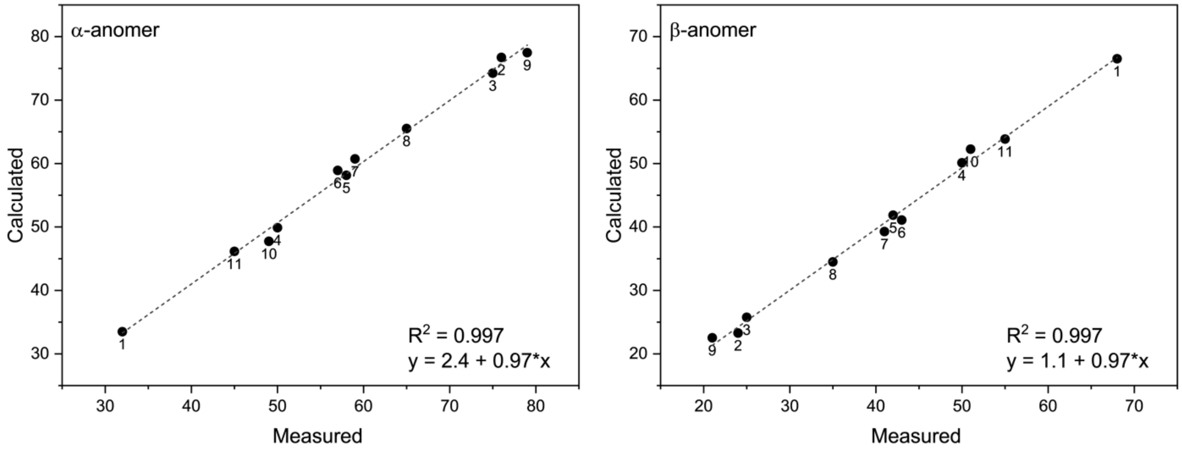

Figure S29. Linear regression of calculated vs. measured anomeric compositions (obtained with NMR) of the mixture samples.

For the SRS quantification, 5–7 images were captured (single Z-planes, sized 141x141 μm^2) at randomly selected locations. An example image from each mixture sample is shown in **Figure S30**. Additionally, five control images of a cover glass without any sample (blank) were measured to confirm that the quantification accurately yielded 0% for all forms. Regression analysis (excluding the blank) comparing the real concentrations with the SRS quantification results (**Figure S31**) indicated that, LASSO and area-based quantification performed slightly better than CLS, based on the R^2 values. LASSO was more accurate due to its ability to reduce overfitting, although some overfitting was still apparent, as pure forms still showed traces of other forms. In the area-based quantification, each pixel was classified to a single compound (the one with the highest score) before calculating the areas.

SRS quantification shows visibly larger errors compared to the XRPD analysis shown in **Figure S28**. This discrepancy was anticipated, given that XRPD, as a bulk method, is inherently less sensitive to inhomogeneities within mixtures. Visual inspection of the mixture images (**Figure S30**) revealed that completely homogeneous mixtures were not achieved; for instance, the β-ANH appeared unevenly distributed. While the limited number of imaged areas is a challenge, it is not the sole factor; another likely contributor is segregation (in the Z-direction) due to particle size and morphology. α-MNH, despite sieving, had a particle size larger than other materials, leading to its probable underrepresentation in the images as smaller particles likely settled at the bottom. Additionally, particle morphology likely influenced the results; α-ANH, which appears as flat crystals, likely oriented in a manner that maximized surface exposure in the images, thus biasing the quantification towards higher concentrations. Conversely, the spray-dried amorphous particles, characterized by their round shape, smooth surfaces, and relatively uniform size distribution, exhibited good flow properties and appeared to mix most homogeneously, which could explain why this form showed the lowest error.

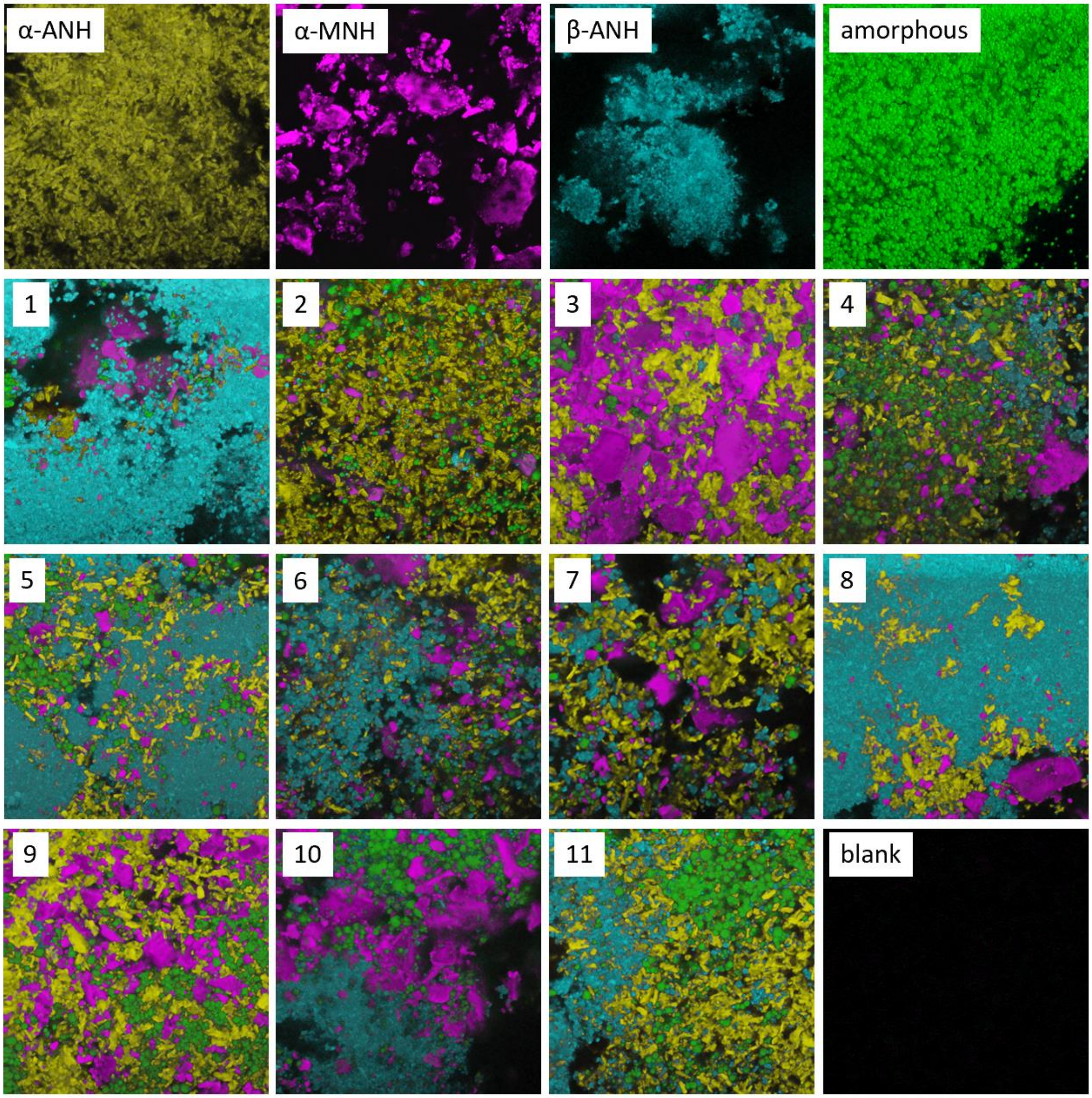

Figure S30. Randomly selected examples of SRS images of the mixture samples and a control image of a cover glass without any sample (blank) (sized 141x141 μm^2).

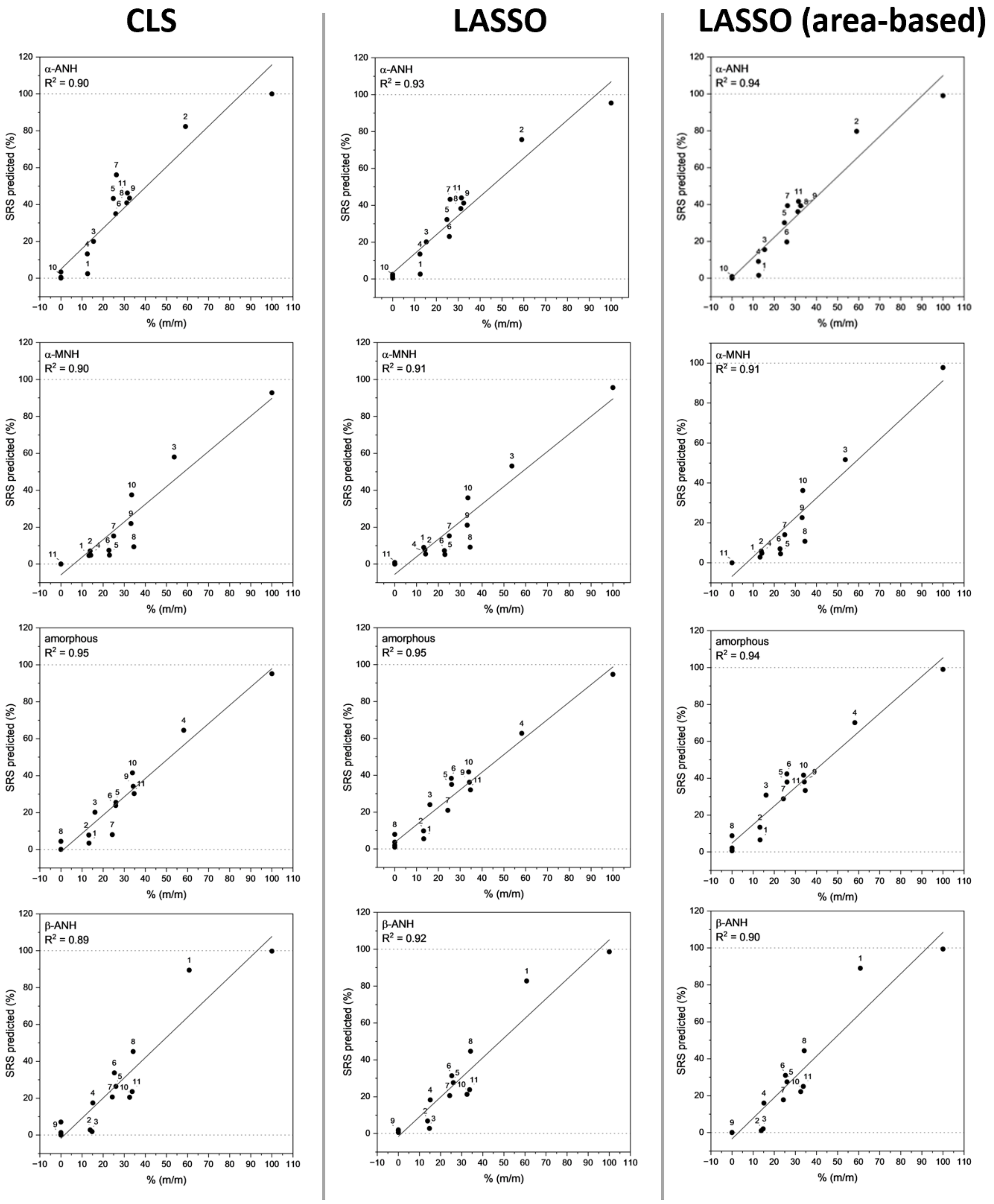

Figure S31. Linear regression of SRS quantification vs. real compositions of the mixture samples (labelled with numbers shown in Table S4).

CLS-based quantification of the commercial samples

The quantitative results from CLS unmixing for the commercial samples are shown in **Table S6**. The results show that with CLS, the concentrations for minor trace forms are considerably higher, while concentrations of main forms decrease when compared to LASSO results. In the qualitative inspection of XRPD diffractograms (**Figure S22, Figure S27**) α-MNH was detected in Lactohale® 400 (LH) and SuperTab® 24AN (STAN), and β-ANH in Lactopress® Granulated (LPG), with no other trace forms found. Overall, the XRPD results align better with LASSO quantification than with CLS. For instance, in the Lactopress® Granulated sample, the α-ANH concentration using CLS is 8.5%, whereas in XRPD this form was undetectable.

Table S6. Summary of SRS quantification results, obtained with CLS unmixing. Values represent the range (minimum–maximum concentration) across the 5 Z-stacks. The means are indicated in bold in parentheses.

	LH	LH, con	STSD	STSD, con	STAN	LPG
**α–ANH**	8.6–13.2 **(10.3)**	14.5–21.9 **(17.1)**	5.6–15.9 **(10.2)**	13.8–20.9 **(17.7)**	8.7–11.9 **(9.7)**	6.5–10.3 **(8.5)**
**α–MNH**	4.2–6.0 **(5.3)**	0.2–0.7 **(0.4)**	60.6–65.2 **(62.3)**	60.6–63.3 **(62.3)**	5.7–11.1 **(8.3)**	68.9–88.3 **(78.2)**
**Amrp**	2.6–5.0 **(3.7)**	1.6–4.0 **(2.9)**	17.5–22.7 **(19.9)**	1.5–4.2 **(3.2)**	0.5–1.4 **(0.9)**	1.1–2.9 **(1.8)**
**β–ANH**	78.4–82.4 **(80.8)**	75.9–82.5 **(79.6)**	1.9–13.4 **(7.6)**	12.1–24.1 **(16.9)**	79.1–84.1 **(81.1)**	3.6–20.4 **(11.6)**

Abbreviations: LH=Lactohale® 400; STSD=SuperTab® 14SD; STAN=SuperTab® 24AN; LPG=Lactopress® Granulated; Amrp=amorphous; ND=not detected; con=conditioned

Limit of detection

In bulk spectroscopy, determining the detection limit (LOD) is comparatively straightforward. The LOD can be established where the signal is three times greater than the standard deviation of noise, among other approaches defined for example, by the International Council for Harmonisation of Technical Requirements for Pharmaceuticals for Human Use (ICH) guidelines[1], and this can be experimentally validated by analyzing samples with concentrations around the LOD value. However, in SRS, determining the LOD for solid mixtures experimentally is challenging, when determined for each pixel (single spectrum) rather than the entire image. An experimental approach would require a sub-pixel level dilution series of different lactose forms, and preparation of such samples is not possible.

Therefore, the LODs were estimated by comparing the single pixel SRS signal strength for a pure analyte against the background noise. A lactose mixture containing β-ANH, α-ANH, α-MNH, and SD lactose was analyzed, and from this image the strongest pixels for each lactose form (where the sample is well focused) were identified. The selected pixels were used to estimate the maximum signal intensity for each lactose form. The false-color image and the chosen pixels are shown in **Figure S32** together with the pixel spectra. Using the criterion that a detectable

[1] International Conference on Harmonisation of Technical Requirements for Registration of Pharmaceuticals for Human Use. **2005**, Validation of analytical procedures: Text and methodology Q2(R1)

sample spectrum must have a peak value at least three times the standard deviation of the background spectrum, the LOD values for each component were derived (**Table S7**). Considering the possible errors in the estimates, it is possible to conclude that the single pixel detection limits are approximately 5%.

Although the single pixel detection limits determined are relatively modest, SRS and SFG microscopy-based analysis allows detection of trace contaminant particles well below these detection limits. As an example, it was found out that the α-ANH reference sample prepared in the laboratory actually contained some very small amounts of a contaminant only detectable by SRS microscopy (**Figure S33**), and not XRPD. Specifically, within a series of 25 images of α-ANH, two particles of a compound were identified, totaling to an area-based concentration of 0.002%. This is an approximately 500 to 1000-fold lower concentration than typically achievable with standard solid-state methods. The exceptionally low detection limit of nonlinear optical microscopy in pharmaceutical solid-state analysis has also been discussed by Schmitt et al. (2015)[2], in their study where they used SFG to detect trace crystallinity in binary mixtures.

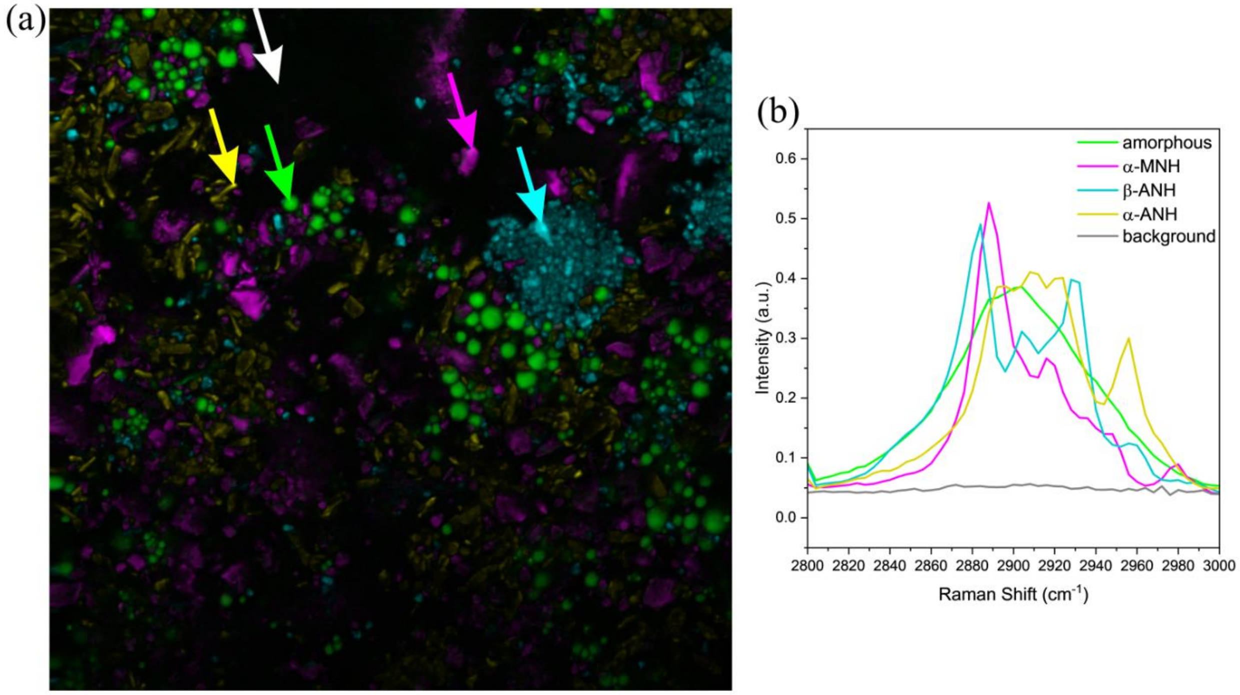

Figure S32. (a) SRS image of the lactose mixture containing β-ANH, α-ANH, α-MNH, and SD lactose. The arrows indicate pixels where the spectra in (b) were extracted. These spectra were used for deriving LOD values for each of the four components.

[2]Schmitt, P. D.; Trasi, N. S.; Taylor, L. S.; Simpson, G. J.; Finding the needle in the haystack: characterization of trace crystallinity in a commercial formulation of paclitaxel protein-bound particles by Raman spectroscopy enabled by second harmonic generation microscopy. *Mol. Pharm.* **2015**, 12(7), 2378-2383.

Table S7. Single pixel detection limit estimates for β-ANH, α-ANH, α-MNH, and amorphous lactose (%, rounded to nearest whole number). The values were approximated as three times the standard deviation of the background spectrum.

**Compound**	**Single pixel detection limit estimate (%)**
α-MNH	3
α-ANH	4
β-ANH	3
Amorphous	4

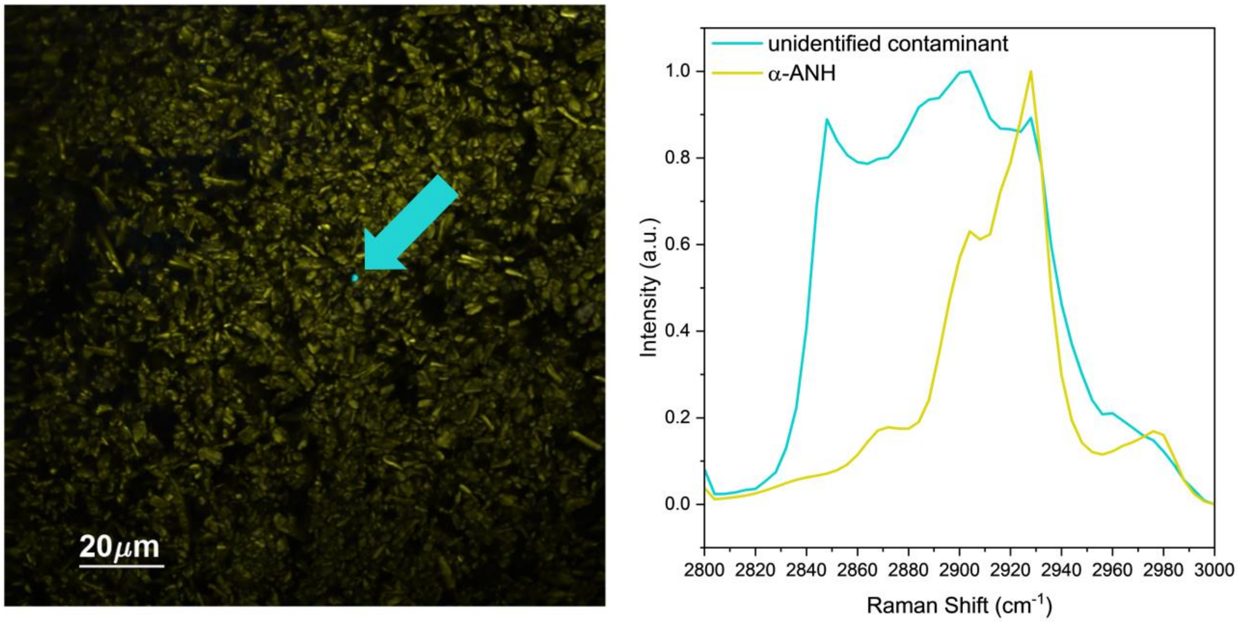

Figure S33. SRS image of α-ANH showing trace particle of an unidentified contaminant (indicated by the arrow), and spectrum of the contaminant and α-ANH.

Quantification error due to limited number of images

To evaluate the error introduced by an insufficient number of images, a large number of images was recorded from mixture samples #3 and #9 (see **Table S4**). The distribution of quantified compositions was then analyzed from individual images and it was assessed how deviations reduced when multiple images were averaged.

Figure S34 shows violin plots of 25 images from each of the two mixture samples (different dataset than that used for the images in **Figure S30**). For sample #3, the area-based mean concentration value (horizontal colored line) differs from the mass fraction, particularly for β-ANH. For sample #9, α-MNH is clearly underrepresented, which is likely due to its larger particle size. These discrepancies are due to sample inhomogeneity/segregation, which cannot be fully avoided by increasing the sampled area.

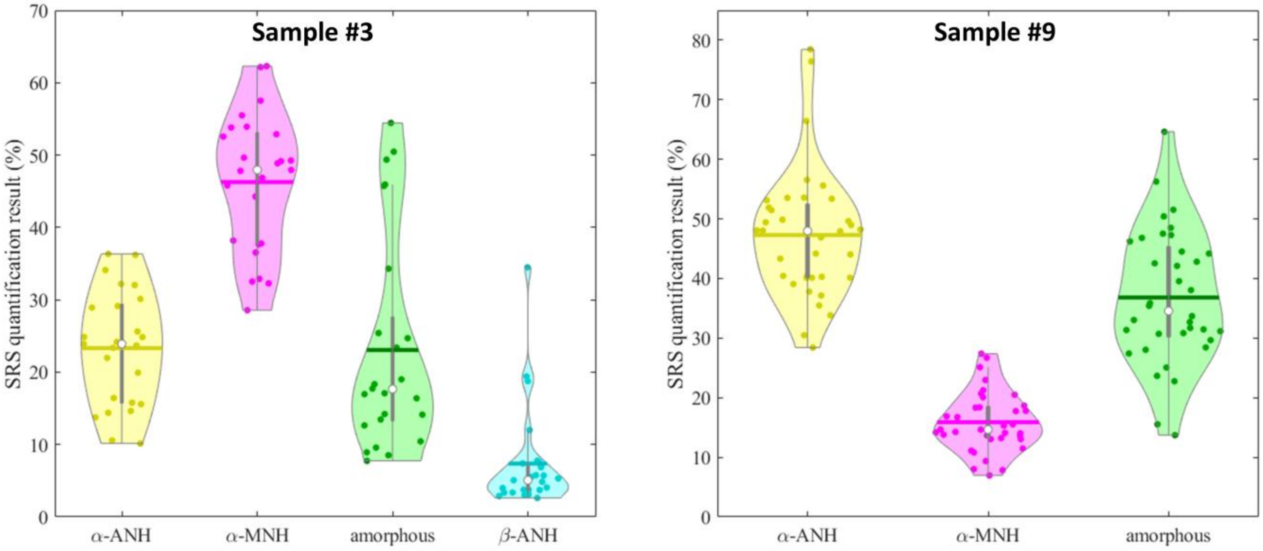

Figure S34. Violin plots showing quantification results (area-based concentration) for 25 images recorded of the mixture samples #3 and #9. The horizontal colored lines represent the mean value of the whole dataset, white dot is the median, and gray bar is the interquartile range.

To further explore how the analyzed sample area affects the quantification, the standard deviation of quantification results was plotted as a function of the number of images averaged. To avoid selection bias in averaging subsets, all possible combinations of image subsets were computed using MATLAB's *nchoosek* function. The number of different sub combinations was therefore the binomial coefficient, defined as

$$ {}_nC_k = \binom{n}{k} = \frac{n!}{(n-k)!\,k!} $$

The solid lines in **Figure S35** show an example trace for one subset combination. The number of recorded images per sample in other parts of the study was five. With five averaged images, the graph shows that the standard deviation was approximately ±5%, which was interpreted as the approximate error caused by the limited number of images acquired.

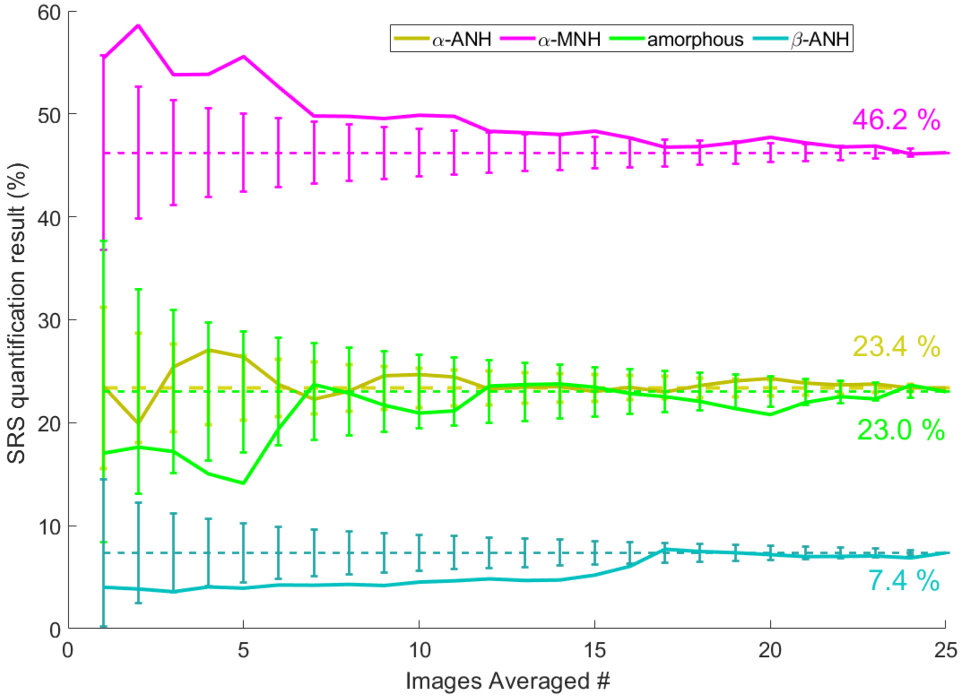

Figure S35. The change of standard deviation of the quantification results (sample #3) as function of number of images averaged. Dashed lines and the values shown on the plot represent the mean quantification results of the 25 images, while the error bars indicate the standard deviation between results from different image subsets. The horizontal axis shows the number of images averaged together, with solid lines showing an example trace for one subset combination.
